# Time‐Resolved SNOM via Phase‐Domain Sampling

**DOI:** 10.1002/nap2.70178

**Published:** 2026-06-22

**Authors:** Philipp Schwendke, Julia Stähler, Samuel Palato

**Affiliations:** ^1^ Institut für Chemie Humboldt‐Universität zu Berlin Berlin Germany; ^2^ Abteilung Physikalische Chemie Fritz‐Haber‐Institut der Max‐Planck‐Gesellschaft Berlin Germany

**Keywords:** phase‐domain sampling, pseudo‐heterodyne, SNOM, time‐resolved, WS_2_

## Abstract

Time‐resolved scanning near‐field optical microscopy (tr‐SNOM) enables the measurement of the dynamic optical response of functional surfaces beyond the diffraction limit. Experimental challenges are imposed both by the use of a pulsed light source and by the need for interferometric signal modulation to isolate the near‐field contribution. We present a novel experimental approach to retrieve the tr‐SNOM signal using a 200‐kHz laser system and pseudo‐heterodyne modulation. We circumvent the Nyquist limit for spectral demodulation by sampling modulation phases, pump intensity, and SNOM signal for every laser shot. A general time‐resolved SNOM signal is derived, independent of the detection scheme or physical assumptions about the near‐field enhancement, and is successfully measured and isolated on WS_2_ monolayer and multilayer regions. We confirm localization by signal–distance curves, spatial confinement at material boundaries, and by identifying signal contributions at individual modulation harmonics. Disentangling the dynamic contributions enables us to extract the dynamic dielectric function of the sample. Showing the capability of phase‐domain sampling paves the way to the integration of more diverse and specialized light sources, growing the potential of optical ultrafast near‐field measurements.

## Graphical abstract

Time‐resolved scanning near‐field optical microscopy (tr‐SNOM) grants access to the dynamic dielectric function of monolayer and multilayer WS_2_, using visible wavelengths. In order to use a femtosecond laser with a repetition rate of 200 kHz, the signal is sampled and demodulated in the modulation phase domain, marking the first realization of tr‐SNOM using sparse sampling in the time domain.

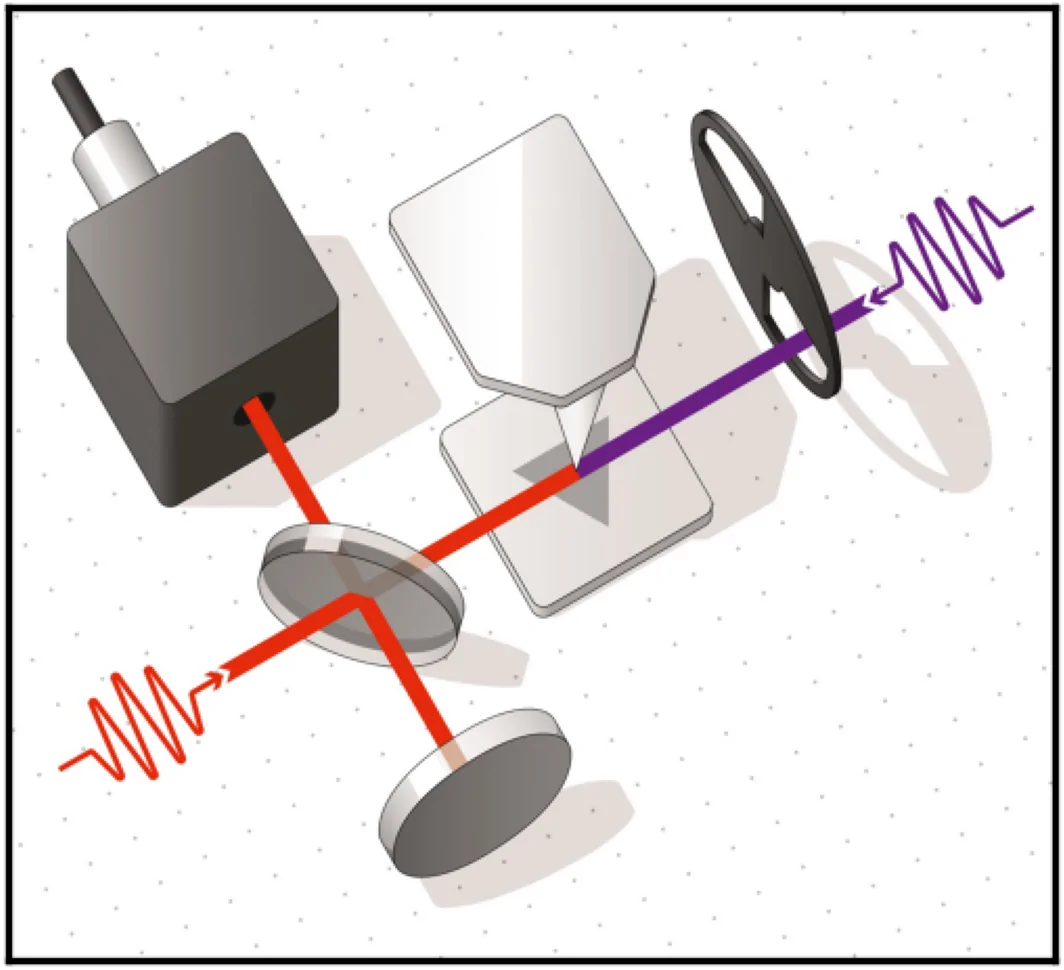

## 1 Introduction

The unique potentials of functional materials, such as 2D materials and their heterostructures, emerge from their electronic structure on the nanoscale. Scanning probe microscopy is uniquely suited to investigate a large range of material properties, offering simultaneous topographic, optical, mechanic, and electric information [1, 2], in a *lab on a tip* approach [3]. Light–matter coupling is especially interesting on length scales comparable to or below the wavelength of the light, with direct technological applications [4, 5], but also unique physics, for example, nano‐resonators [6] and polaritons [7]. Although scanning near‐field optical microscopy [8] (SNOM) pushes the spatial resolution beyond the diffraction limit, ultrafast measurements offer insights into functional processes via signal kinetics. In a combination, time‐resolved SNOM (tr‐SNOM) has been used to characterize carrier dynamics in semiconductors [9, 10], follow the insulator‐to‐metal transition in VO_2_ [11, 12, 13], and describe exciton dynamics in WS_2_ [14, 15].

However, ultrafast measurements require a pulsed light source, limiting the sampling frequency by its repetition rate. It is necessary to resolve pump modulation simultaneous to higher orders in tapping modulation [16] and phase modulation when probing in the visible [17]. Consequently, tr‐SNOM was either limited to MHz repetition rate laser systems [9, 10, 14], or to using AFM tips with a lower resonance frequency [18]. Introducing phase‐domain sampling, Wang et al.. [19] were able to circumvent the Nyquist limit in sampling frequency for a self‐homodyne (shd) detection scheme. This extends the possible implementation of SNOM to kHz laser systems, providing stable high peak powers useful, for example, for parametric amplification, white light generation, photo‐switching, or nonlinear spectroscopy. Phase‐domain sampling was extended to pseudo‐heterodyne modulation (pshet) [20] by sampling modulation phases independently for every laser shot using quadrature‐assisted discrete (QUAD) demodulation [21]. However, tr‐SNOM using phase‐domain sampling has yet to be demonstrated.

In this paper, we extend QUAD sampling to ultrafast pump–probe measurements. Using a 200‐kHz laser system, we present the first realization tr‐SNOM acquired in the phase domain, completely independent of the sampling frequency. First, we derive a tr‐SNOM signal from basic principles that is independent of any physical model of the near‐field interaction. Our new experimental approach retrieves the static and excited SNOM signal simultaneously and independently after a cascaded demodulation scheme. We are able to distinguish background and near‐field contributions in the harmonic orders of the tr‐SNOM signal and confirm the spatial confinement in a WS_2_ monolayer/multilayer boundary region. By isolating and quantifying the dynamic tip response, we model the near‐field response of WS_2_ and obtain a dynamic dielectric function.

## 2 A General Time‐Resolved SNOM Signal

The essence of optical pump–probe, or any ultrafast measurement, is to compare a static signal (*pump‐off*) to a dynamic signal a certain time after excitation (*pump‐on*). We now define a time‐resolved SNOM signal based on these two measurements.

In static SNOM, we measure the electric field scattered by the tip–sample junction $E_{\text{sca,}\;\text{off}}=\sigma_{\text{off}}E_{0}$, described by the scattering coefficient σ and the incident light $E_{0}$. The scattered light consists of optical near field (NF) and a background signal (BG): $\sigma_{\text{off}}=\sigma_{\text{NF}}+\sigma_{\text{BG}}$. The experimental challenge in SNOM is to isolate $\sigma_{\text{NF}}$. Detecting visible wavelengths, this is efficiently achieved by demodulating high harmonics of tapping modulation and introducing a local oscillator $E_{\text{ref}}$ for interferometric detection. The photodiode measures the combined intensity of NF, BG, and reference signal (ref).

(1) $$|E_{\text{sig,off}}{|}^{2}=|E_{\text{NF}}+E_{\text{BG}}+E_{\text{ref}}{|}^{2}$$

(2) $$=|E_{\text{NF}}{|}^{2}+|E_{\text{BG}}{|}^{2}+|E_{\text{ref}}{|}^{2}+E_{\text{NF}}E_{\text{BG}}^{*}+E_{\text{ref}}\left(E_{\text{NF}}^{*}+E_{\text{BG}}^{*}\right)+c.c.$$

where $E_{\text{NF}}$ and $E_{\text{BG}}$ are the near‐field and background contributions to the detected signal. Pshet modulation employs a periodic modulation of the reference phase, thereby allowing the isolation of $\sigma_{\text{off}}\propto E_{\text{NF}}+E_{\text{BG}}$ in the signal spectrum [20]. Demodulation at harmonics of AFM tapping modulation then suppresses $E_{\text{BG}}$ [22, 23]. Without a local oscillator, the SNOM signal is dominated by the term $E_{\text{NF}}{E_{\text{BG}}}^{*}$, containing a multiplicative background contribution in all harmonics [20].

Optical pump–probe measurements are a third‐order nonlinear process [24] and as such an addition to the static signal. The modified material response after photo‐excitation by the pump pulse leads to additional time‐dependent contributions in both NF and BG signals: $\Delta E\left(t\right)=\Delta E_{\text{NF}}\left(t\right)+\Delta E_{\text{BG}}\left(t\right)$. The reference field does not interact with the sample and therefore is not modified by the pump. The total signal at the detector is as follows:

(3) $$\begin{array}{ll} E_{\text{sig,on}} & =E_{\text{sig,off}}+\Delta E \end{array}$$

(4) $$\begin{array}{ll} |E_{\text{sig,on}}{|}^{2} & =|E_{\text{sig,off}}{|}^{2}+|\Delta E_{\text{NF}}{|}^{2}+|\Delta E_{\text{BG}}{|}^{2}+\Delta E_{\text{NF}}\left(E_{\text{NF}}^{*}+E_{\text{BG}}^{*}+\Delta E_{\text{BG}}^{*}\right)\\ & \;+\Delta E_{\text{BG}}\left(E_{\text{NF}}^{*}+E_{\text{BG}}^{*}\right)+E_{\text{ref}}\left(\Delta E_{\text{NF}}^{*}+\Delta E_{\text{BG}}^{*}\right)+c.c. \end{array}$$

As in the static case, pshet modulation isolates the contribution $\sigma_{\text{off}}+\Delta \sigma \left(t\right)$. Subtracting the static signal yields the pump‐induced change in scattered light intensity.

(5) $$\Delta \sigma \left(t\right)=\sigma_{\text{on}}\left(t\right)-\sigma_{\text{off}}$$

Using self‐homodyne modulation (shd), $E_{\text{ref}}=0$, and the dynamics of $\Delta E_{\text{NF}}\left(t\right)$ are overshadowed by $\Delta E_{\text{BG}}\left(t\right)$ in terms such as $\Delta E_{\text{BG}}\left(t\right)E_{\text{NF}}^{*}$, which is the interference between the spatially resolved static near‐field and the time‐dependent background. The effect has initially been demonstrated by imaging on VO_2_ films [18] and is further demonstrated theoretically and experimentally by time‐resolved measurements provided in the Supporting Information S1. The dominance of multiplicative background is completely analogous to the static case, where it was shown that even the higher order NF is modulated by the far‐field background signal [20]. To isolate the time‐resolved near‐field signal, it is therefore necessary to employ a triple modulation scheme: tapping modulation, interferometric detection, and pump modulation. This enables the measurement of Δσ(t) in amplitude and phase.

To illustrate pump–probe modulation in tr‐SNOM and to help define signal normalization, we will be using a phasor analysis approach. Figure 1a visualizes Equation (5). $\sigma_{\text{off}}$ is the pshet signal acquired before photo‐excitation. It is demodulated at tapping harmonic n=4, suggesting dominance of $E_{\text{NF}}$. $\sigma_{\text{on}}\left(t\right)$ is the pshet signal acquired after photo‐excitation and is dominated by $\Delta E_{\text{NF}}+E_{\text{NF}}$ when demodulated at higher tapping harmonics. The difference Δσ(t) is then proportional to $\Delta E_{\text{NF}}$.

**FIGURE 1:**
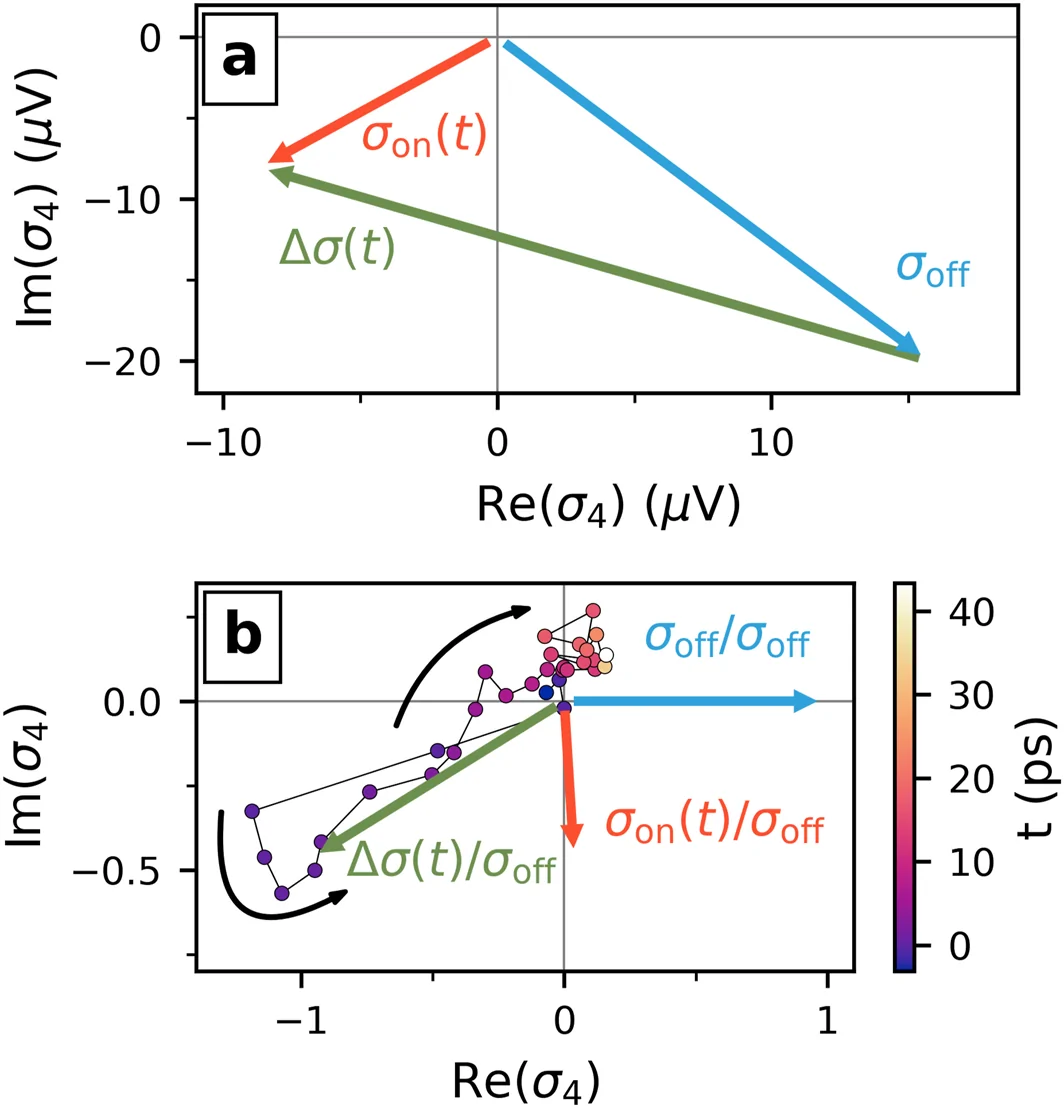
Visualization of the complex SNOM signal in a pump–probe measurement. (a) $\sigma_{\text{on}}$ is the signal after pump excitation (*pump‐on*), $\sigma_{\text{off}}$ is the SNOM signal without pump excitation (*pump‐off*), Δσ is the pump‐induced difference of the two. (b) All signals normalized by the probe‐only signal $\sigma_{\text{off}}$. Example experimental time‐resolved SNOM signal shown as colored circles. Pump–probe time delay is indicated by black arrows and the colorbar on the right.

Although isolating $\Delta \sigma_{\text{NF}}\left(t\right)$ already yields the time‐resolved near‐field signal, its magnitude and phase are dependent on those of $\sigma_{\text{off}}$, which are determined by experimental factors that are in general not known and might drift during the measurement. In order to avoid these effects and to recover the sample material response, we normalize the signal by $\sigma_{\text{off}}$, as illustrated in Figure 1b. This has the effect to rotate and scale all the phasors. $\sigma_{\text{off}}/\sigma_{\text{off}}$ becomes unity by definition. $\sigma_{\text{on}}\left(t\right)/\sigma_{\text{off}}$ describes the relative amplitude and the difference in phase of *pump‐on* and *pump‐off* signals. The time‐resolved SNOM signal is then

(6) $$\frac{\Delta \sigma \left(t\right)}{\sigma_{\text{off}}}=\frac{\sigma_{\text{on}}\left(t\right)-\sigma_{\text{off}}}{\sigma_{\text{off}}}=\frac{\sigma_{\text{on}}\left(t\right)}{\sigma_{\text{off}}}-1$$

as shown by the green arrow.

This normalization has a few advantages. It does not depend on physical assumptions about the tip–sample interaction. The ratio $\Delta \sigma \left(t\right)/\sigma_{\text{off}}$ cancels arbitrary signal scaling and phase shifting due to experimental geometry. This signal is therefore not distorted by experimental factors such as the intensity of the probe pulse, quality of the interferometric contrast, alignment to the AFM tip, magnitude of NF signal, or arbitrary optical phase offset, nor by their drifts. The magnitude and phase of Δσ(t)/σ can be interpreted directly. For example, a phase of π indicates a pump‐induced reduction in signal amplitude, whereas a phase of 0 corresponds to an increase. $\Delta \sigma \left(t\right)/\sigma_{\text{off}}$ also provides ways to assess the validity of the data: causality imposes $\Delta \sigma \left(t\right)/\sigma_{\text{off}}=0$ for t<0. The signal must also go to 0 when the pump is blocked, and the magnitude of $\Delta \sigma \left(t\right)/\sigma_{\text{off}}$ allows the optimization of the pump–probe overlap. The signal should also vanish scattering off of media that do not interact with the pump, such as fused silica in the visible. Furthermore, the ratio $\sigma_{\text{on}}/\sigma_{\text{off}}$ allows the compensation of probe beam intensity fluctuations, thereby improving the signal‐to‐noise ratio.

The black dots in Figure 1 show experimental measurements of $\Delta \sigma \left(t\right)/\sigma_{\text{off}}$, with the direction of time indicated by black arrows. At negative times, the signal is initially around the origin. At t=0, it suddenly increases in amplitude and moves to the lower left quadrant. Then, the pump–probe signal decays to a longer lived component with a phase ∼π/2, decaying to 0 as $\sigma_{\text{on}}\left(t\right)$ approaches $\sigma_{\text{off}}$ for large t. This is the expected behavior for a pump–probe signal that obeys causality. The tr‐SNOM signal will be referred to as Δσ/σ in the text below.

## 3 Methods

Our tr‐SNOM setup consists of a commercial SNOM (neaSNOM, neaSpec) and a 200‐kHz femtosecond laser (PHAROS, Lightconversion) driving two independent optical parametric amplifiers (ORPHEUS, Lightconversion) for pump and probe pulses. Figure 2a shows a scheme of the optical setup. The pump pulse enters a delay stage and is chopped using a chopping wheel, the status of which is detected with a photodiode for every laser shot. This assignment enables the simultaneous acquisition of $\sigma_{\text{on}}$ and $\sigma_{\text{off}}$ during the integration time, reducing low frequency noise contributions. The probe pulse is spatially filtered (not shown) and enters a Michelson interferometer with the sample in the signal arm and a vibrating mirror in the reference arm for established pshet modulation. Pump beam and signal arm are combined in a parabolic mirror and focused on an AFM tip operating in tapping mode. The parabolic mirror reflects the light scattered at the tip–sample junction back into the interferometer. Following the pshet modulation scheme described by Ocelic et al.. [20], the interference of signal and reference arm is detected at the photodiode.

**FIGURE 2:**
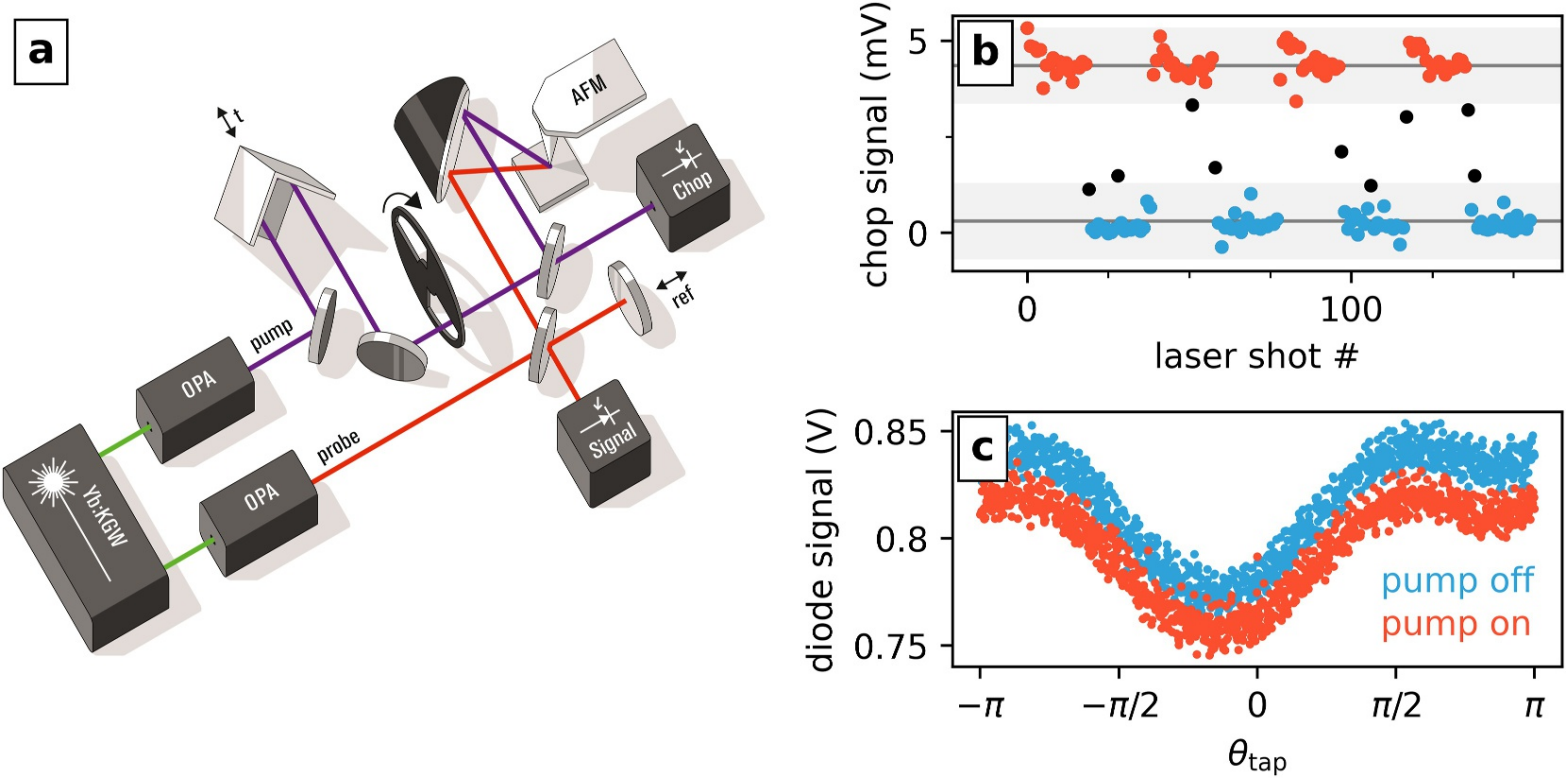
(a) Schematic of the time‐resolved SNOM optical setup. The pump beam path is delayed with respect to the probe beam path and chopped using an optical chopper wheel. The state of the pump is detected using the “chop” photodiode. The probe beam path enters a Michelson interferometer for pseudo‐heterodyne modulation. Graphic by Peter Schwendke. Reproduced with permission. (b) Photodiode voltage determining the state of the chopper wheel. Red: *pump‐on*, blue: *pump‐off*, black: pump pulses clipped by the chopper wheel are disregarded during analysis. (c) Comparison of the voltage of the photodiode after optical excitation (*pump‐on*) and without (*pump‐off*). The intensity of individual laser pulses shows tapping modulation with multiple harmonics and a pump‐induced change in signal level.

In order to measure the tr‐SNOM signal, we extend the QUAD stroboscopic scheme as follows. [21]. The continuous modulation signals, that is, deflection laser and piezo voltage of the reference arm, are filtered and used to generate two copies: one in‐phase (x) and one with a π/2 phase shift (y). x and y are effectively cosine and sine of the modulation phase, which can then be recovered as θ=arctan2(y/x). This calculation is performed for both the tapping and reference arm modulation, yielding $\theta_{\text{tap}}$ and $\theta_{\text{ref}}$. As in the static case, the two (x,y) signal pairs are detected using a data acquisition card, alongside the voltage at the signal photodiode. For time‐resolved SNOM, we add one detection channel: the status of the chopping photodiode. The acquisition of all channels is synchronized using a 200‐kHz sampling clock obtained from the laser amplifier. Experimental control, signal acquisition, and analysis are accomplished using dedicated python scripts, which are fast enough for live usage [25].

To acquire $\sigma_{\text{on}}$ and $\sigma_{\text{off}}$ simultaneously, detected tuples of the photodiode signal $\propto |E_{\text{sig}}{|}^{2}$, tapping modulation phase $\theta_{\text{tap}}$, and reference modulation phase $\theta_{\text{ref}}$ are first separated based on the chopping state. Figure 2b shows the voltage of the chopping photodiode for consecutive laser shots. The chop signal has two mean values marked by grey lines: near 0 when the pump pulse is blocked by the chopper, and ∼4.5 mV when the pump pulse passes and reaches the AFM tip. The two cases are selected as quantiles over a set of samples, acquired during the integration time. The first analysis step in demodulating the time‐resolved signal is separating *pump‐on* and *pump‐off* tuples and neglecting shots where the pump is clipped by the chopper wheel.

Figure 2c shows the photodiode signal in the tapping phase domain, measured for individual laser shots. The *pump‐off* signal shows only the detected probe pulses where the pump was blocked. The *pump‐on* signal shows only the detected probe pulses where the pump was not blocked. Demodulation of the separated data points gives access to $\sigma_{\text{off}}$ and $\sigma_{\text{on}}\left(t\right)$, respectively.

To recover the pshet modulated time‐resolved signal, both subsets of the data undergo identical demodulation steps. The demodulation algorithm is slightly improved with respect to our previous work [21]. First, all samples of the photodiode signal are averaged in a binned 2D phase domain spanned by $\theta_{\text{tap}}$ and $\theta_{\text{ref}}$. This creates an array of real values with tapping modulation along $\theta_{\text{tap}}$ and pshet modulation along $\theta_{\text{ref}}$. Performing a fast Fourier transform along $\theta_{\text{tap}}$ gives us access to the harmonics of tapping modulation.

(7) $$|E_{\text{sig}}{|}^{2}=\sum_{n}\tau_{n}\left(\theta_{\text{ref}}\right)e^{in\left(\theta_{\text{tap}}-\theta_{C}\right)}$$

where $\tau_{n}\left(\theta_{\text{ref}}\right)$ now depends on the tapping demodulation order n and the phase of the reference modulation $\theta_{\text{ref}}$. Describing the motion of the tip as a harmonic oscillation, all harmonics of the tapping modulation are symmetric around the phase of contact $\theta_{C}$. Correcting that phase offset, the harmonic spectrum becomes real and the signal hermitian, that is, $\tau_{n}$ becomes purely real. The imaginary part of $\tau_{n}\left(\theta_{\text{ref}}\right)$, as returned by the fast Fourier transform, can therefore be disregarded as noise.

$\tau_{n}\left(\theta_{\text{ref}}\right)$ describes the pshet modulation, that is, the interference of signal arm and reference arm at the photodiode, as contained in individual tapping harmonics n. The reference arm undergoes an oscillating phase modulation with modulation phase offset $\theta_{0}$ and optical phase offset $\psi_{R}$.

(8) $$E_{\text{ref}}=\rho \;e^{i\psi }E_{0}$$

(9) $$\psi =\gamma \;\sin\left(\theta_{\text{ref}}-\theta_{0}\right)+\psi_{R}$$

ρ is a scaling factor and γ the phase modulation depth in radians. Comparing to Equations (2) and (4), we can separate the contributions of the SNOM signal according to their dependence on $\theta_{\text{ref}}$. All cross terms not multiplied by $E_{\text{ref}}$ and therefore not dependent on $\theta_{m}athrmref$ are collected in the constant $c_{n}$. This isolates the scattering coefficient $\sigma_{n}$.

(10) $$\tau_{n}\left(\theta_{\text{ref}}\right)=c_{n}+E_{\text{ref}}\left(\theta_{\text{ref}}\right)\sigma_{n}E_{0}^{*}+c.c.$$

We assume stability of the reference arm parameters ρ, γ, $\theta_{0}$, and $\psi_{R}$ during the acquisition time. This enables us to fit $\tau_{n}\left(\theta_{\text{ref}}\right)$ with the model function $c_{n}+Re\left(\rho e^{i\psi }\right)$ and attribute the fitting parameters ρ and $\psi_{R}$ to amplitude and phase of $\sigma_{n}$, respectively. γ and $\theta_{0}$ do not depend on the harmonic order and can be fixed fitting $\tau_{0}$ to stabilize the fit for higher harmonics. This procedure is performed on both *pump‐on* and *pump‐off* data points, generating $\sigma_{n,on}$ and $\sigma_{n,o\mathrm{ff}}$. The complex time‐dependent SNOM signal $\Delta \sigma_{n}/\sigma_{n}$ is formed using Equation (6). This demodulation algorithm provides an improved signal‐to‐noise ratio and experimental stability compared to our previous algorithm [21]. The next section will experimentally demonstrate this approach.

## 4 Time‐Resolved Near‐Field Microspopy

We now assess the spatial dependence and kinetics of the tr‐SNOM signal Δσ/σ to confirm our method is able to isolate the time‐resolved NF contribution. A common method to prove the NF character is signal–distance curves, also called retraction or approach curves [26, 27, 28, 29], which we discuss first. In our setup, the AFM tip, as the main scatterer, stays roughly in the same position, whereas the sample is moved downward by the piezo stage. The NF signal $\sigma_{\text{NF}}$, acquired during this retraction, can be distinguished from the background $\sigma_{\text{BG}}$ by its stronger dependence on the tip–sample distance. As the tip–sample distance dz is increased, amplitude and phase of $\sigma_{\text{NF}}$ stay constant, whereas the tip is still in contact. The signal amplitude then decays within the first ∼10nm after losing the tip–sample contact. A longer decay, oscillations, shifting phase, or residual signal indicate a background character that is not necessarily localized at the tip–sample junction. These background signals can be due either to scattering from the sample or from the tip.

Figure 3 shows retraction curves taken on multilayer WS_2_ using an uncoated Si tip. The NF signal is detected at an energy of 1.9 eV, slighty below the A‐exciton resonance of WS_2_, whereas the pump photon energy is 3.0 eV, leading to above bandgap excitation. Figure 3a shows the amplitude of the NF signal $\sigma_{5}$ for both *pump‐on* and *pump‐off*, whereas (b) shows the phase. The acquisition site is marked by a white dot in the AFM topography image of the multilayer region, shown in the inset in (a). Signal amplitude and phase are mostly constant in contact. After the AFM tip snaps off‐contact (dotted line), the *pump‐off* signal (blue) shows a fast amplitude decay with a constant phase, suggesting the NF character. The *pump‐on* signal (red) shows a reduced amplitude in contact, also a rapid amplitude decay, but then a residual signal with a phase that shifts from π/2 to −π with the increasing tip–sample distance. The residual signal is likely a dynamic response by the uncoated Si tip and will be discussed in Section 5.1. The retraction curves are corrected for a constant complex offset at large dz, which we attribute to scattering off of the AFM tip without sample interaction. Retraction curves using PtIr coated tips, as commonly used in SNOM measurements, show similar results, but in general less pump–probe contrast and are presented in Supporting Information S1: Figure S5. The decay of the SNOM signal within ∼10 nm implies its localization at the AFM tip and the dominance of the NF contribution to $\sigma_{\text{on}}$ while in contact.

**FIGURE 3:**
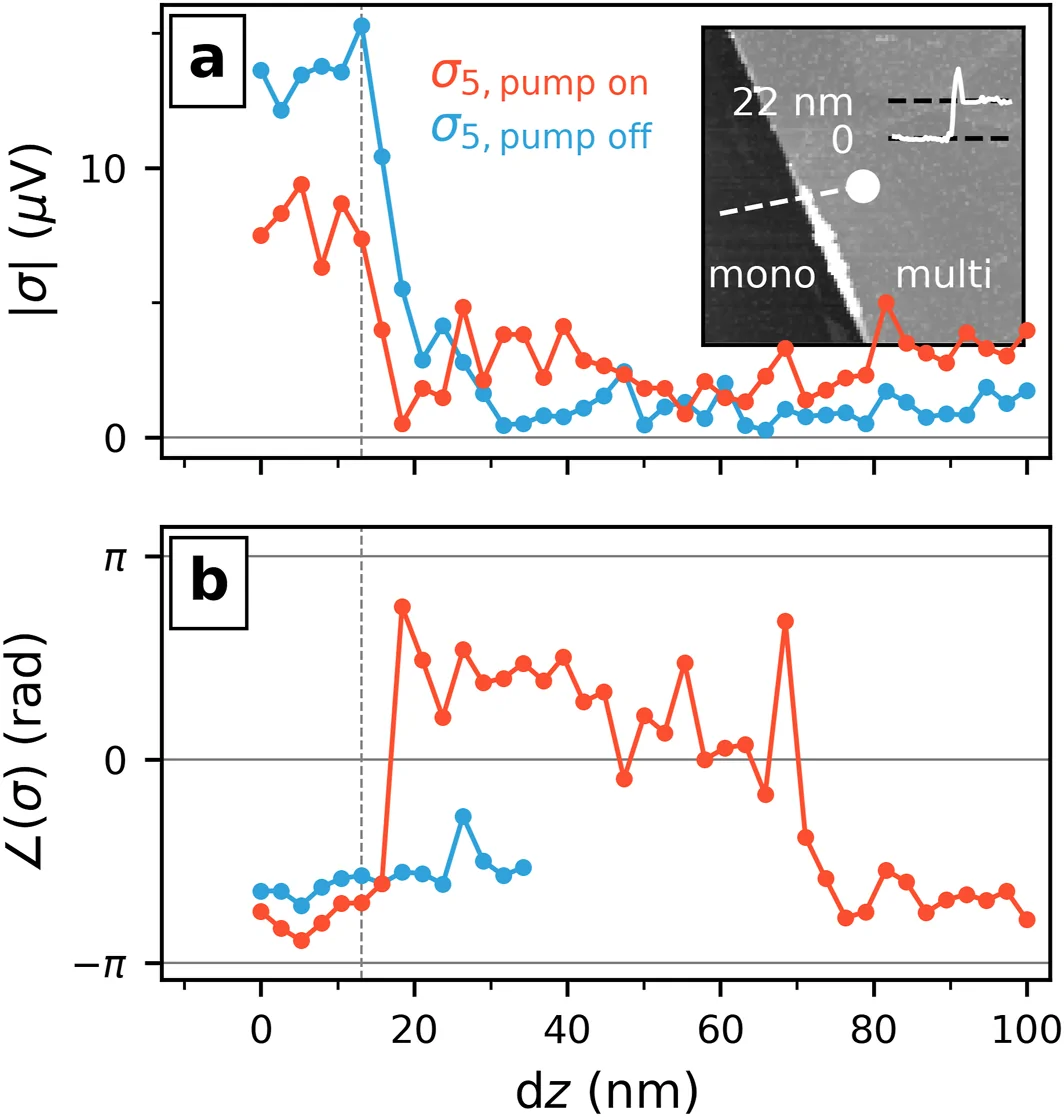
SNOM signal–distance curves taken at the location marked in the AFM inset. (a) Comparison of SNOM signal amplitude $\left|\sigma_{5}\right|$ versus tip–sample distance dz, taken before (*pump‐off*) and 0.5 ps after (*pump‐on*) optical excitation. The dashed line marks the snap‐off distance where the AFM tip loses contact to the sample, defined by the maximum AFM tapping amplitude during retraction. (b) Optical phase of $\sigma_{5}$ with and without optical excitation. At distances where the $\left|\sigma_{5}\right|$ drops below the noise floor, the phase becomes ill‐defined and is not plotted (phase‐blanking [30]). The inset shows an AFM image of the acquisition site with a height profile taken along the dashed line.

To further confirm the identification as pump‐modulated NF, Figure 4 compares the time‐resolved SNOM signal Δσ/σ as a function of pump–probe delay time at various orders. Figure 4a,c shows the amplitude and phase of the lower harmonics, whereas (b) and (d) show the amplitude and phase of the higher harmonics. $\sigma_{0}$ is pure far‐field kinetics, not modulated by AFM tapping, and shows an abrupt rise at t=0, followed by a reduction in SNOM signal amplitude with phase ∼π. The same contribution is visible in $\sigma_{1-3}$ at long times, but a new contribution with a phase close to 2π and a faster time constant is visible in $\sigma_{1}$ and dominates the very early response in $\sigma_{2}$ and $\sigma_{3}$. The kinetics of amplitude and phase appear nontrivial due to the interference of the two complex‐valued contributions, while being a simple addition in the complex plane, similar to the data points plotted in Figure 1b. Finally, $\sigma_{4,5}$ in Figure 4b,d shows a contribution with a phase close to π. The decay is faster than the far‐field kinetics and drops beneath the noise floor after ∼15 ps. All harmonics $\Delta \sigma_{n}/\sigma_{n}$ show no signal at t<0 and rise abruptly at t=0, confirming the isolation of pump‐induced changes.

**FIGURE 4:**
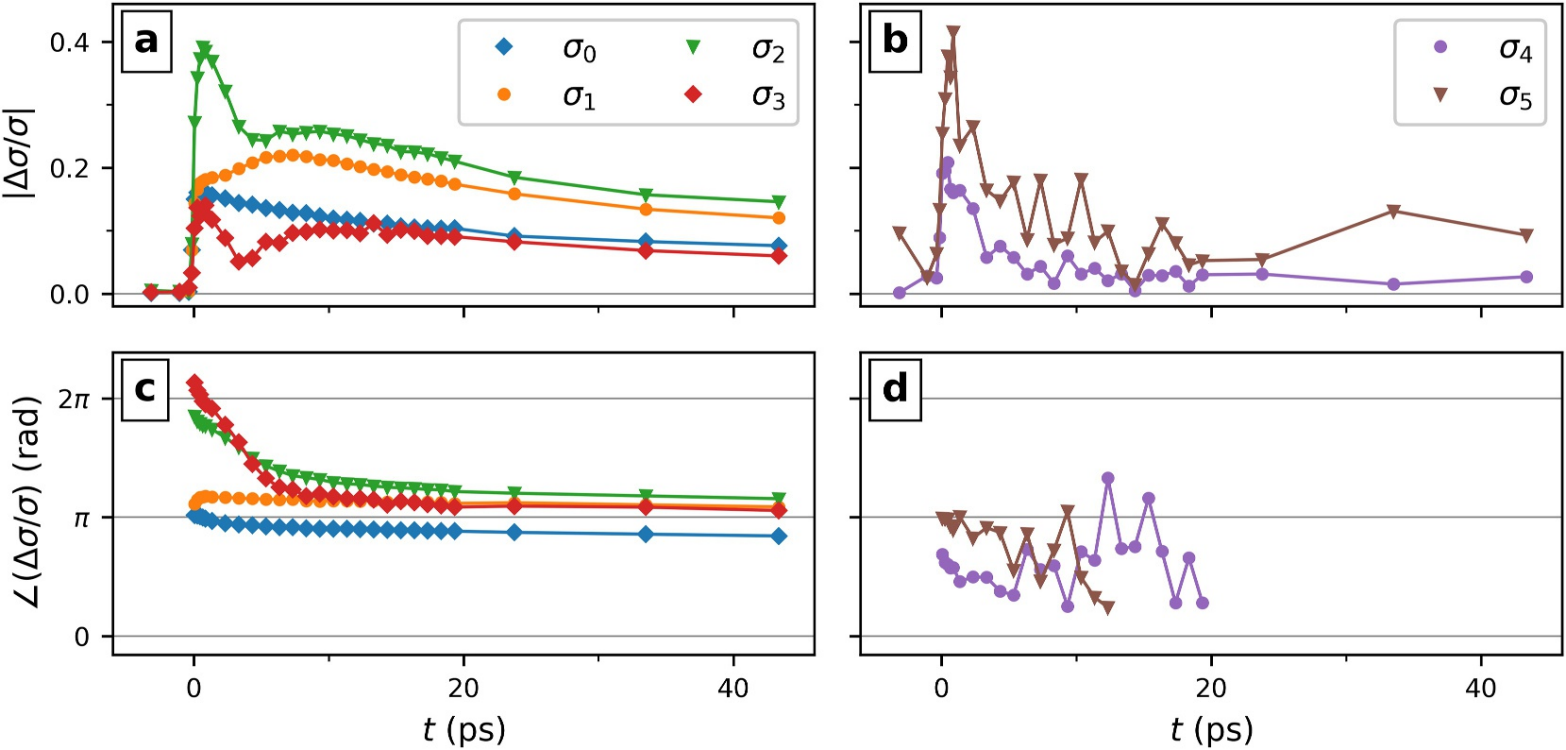
Time‐resolved SNOM signal on multilayer WS_2_ for demodulation order n=0 – 5. (a) and (b) show the signal amplitude versus delay time t, whereas (c) and (d) show the optical phase. $\sigma_{0}$ is the pure far‐field contribution. $\sigma_{1-3}$ shows varying contributions with distinct optical phase and varying amplitudes. $\sigma_{4,5}$ shows a dynamic signal decaying below the noise floor after ∼10 ps, where it is phase‐blanked. Location marked in inset in Figure 3a.

The progressive change of signal contributions is in agreement with the suppression of diffraction‐limited background by higher‐harmonic demodulation. $\Delta \sigma_{0}/\sigma_{0}$ is dominated by $\sigma_{0,BG}$ and $\Delta \sigma_{0,BG}$ and shows pure BG kinetics evolving on long timescales. $\Delta \sigma_{4,5}/\sigma_{4,5}$, on the other hand, is dominated by the NF contribution $\sigma_{4,5,NF}$ and $\Delta \sigma_{4,5,NF}$ showing distinct kinetics. $\Delta \sigma_{1-3}/\sigma_{1-3}$ shows a superposition, as expected. In further analysis, $\sigma_{4}$ will be discussed instead of $\sigma_{5}$ as it is dominated by the same dynamic NF signal, but has an improved signal‐to‐noise ratio.

In contrast, tr‐SNOM acquired without interferometric detection using self‐homodyne (shd) modulation produces the same signal kinetics in all harmonic orders, as shown in Supporting Information S1: Figure S2. Without the use of a reference field $E_{\text{ref}}$, the existence of any BG kinetics $\Delta E_{\text{BG}}$ dominates all kinetic terms in Equation (4). This way, it is not possible to distinguish any tapping harmonic from the far‐field signal. Even in the supposed case that all signal kinetics can be attributed to the NF, for example, by experimental geometry, and setting the question of usefulness of tr‐SNOM in this case aside, the lack of phase information provided by shd modulation makes an unambiguous identification of different contributions impossible. The projection of a superposition of dynamic contributions, which are naturally complex valued, on a real value is not unique, prohibiting direct interpretation.

To confirm the lateral localization of the dynamics observed in $\sigma_{4}$, we compare kinetics at closely spaced locations. Figure 5 shows kinetic measurements taken at different positions across a boundary between WS_2_ monolayer and multilayer regions of the same sample (pos. A, B and C, D respectively). Figure 5a,c shows the gradual change of the background signal $\sigma_{0}$, as the AFM tip moves across the boundary. A contribution with phase ∼π/2 and faster time constant is more dominant on the monolayer, whereas a slower contribution with phase ∼π can be observed on the multilayer.

**FIGURE 5:**
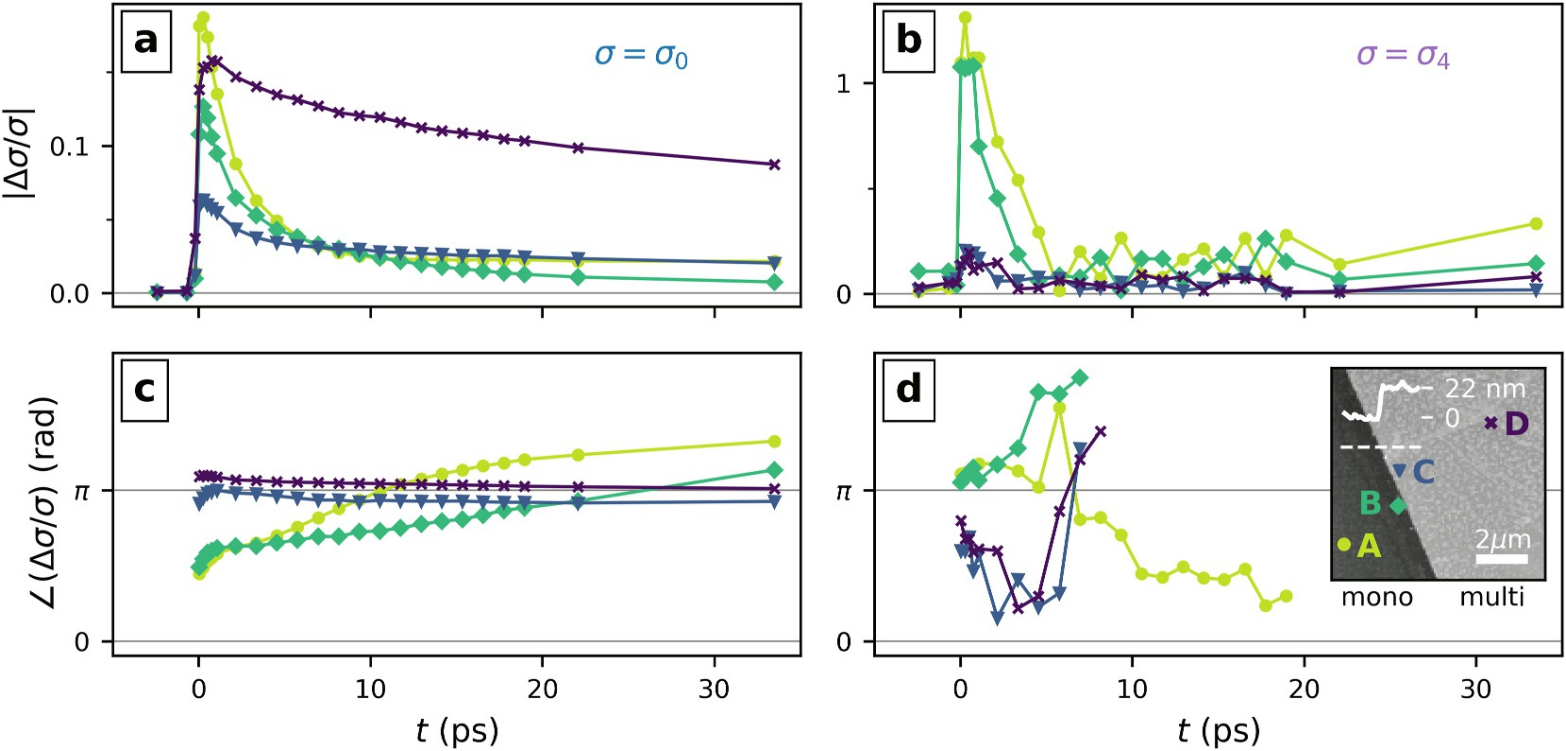
tr‐SNOM signal at different locations on WS_2_ close to a monolayer–multilayer boundary, marked in the AFM inset. (a) and (c) show the amplitude and phase of the pure far‐field contribution $\sigma_{0}$. (b) and (d) show the amplitude and phase of $\sigma_{4}$. Although $\Delta \sigma_{0}/\sigma_{0}$ varies gradually moving across the boundary, $\Delta \sigma_{4}/\sigma_{4}$ shows distinct kinetics on monolayer and multilayer WS_2_. The inset shows an AFM image of the acquisition site with a height profile taken along the dashed line.

$\sigma_{4}$, displayed in Figure 5b,d, features discrete kinetics on monolayer and multilayer regions, each showing similar and reproducible signal amplitude and phase. Interestingly, the pump–probe contrast of $\sigma_{4}$ is enhanced in the monolayer region, suggesting a complete bleach of the NF signal in the first few ps on the monolayer only. In particular, the signals from pos. B and C, located very close to the junction, show a clear contrast. The distinction, even close to the boundary, confirms the localization of the pump–probe signal at the AFM tip.

In summary, the isolation of the time‐resolved NF contribution has been demonstrated in three ways. *Pump‐on* and *pump‐off* signals in Figure 3 show a clear pump modulation together with a strong tip–sample distance dependence, confirming vertical confinement. The kinetics of different harmonic orders in Figure 4 reflect the description in Equation (4): the background dynamics are observed for n=0, and mixed contributions can be identified for n=1 to 3, whereas $4^{th}$ and $5^{th}$ tapping harmonic can be attributed to NF kinetics. The contributions each obey causality for t<0 and show distinct kinetics and optical phase. We were also able to demonstrate high lateral resolution by comparing the tr‐SNOM signal on both sides of a material boundary in Figure 5, showing confinement to the AFM tip.

## 5 Discussion

In the previous sections, we have shown that $\Delta \sigma_{n}/\sigma_{n}$ for sufficiently high n is the time‐resolved NF signal. To present a physical interpretation, we will relate this tr‐SNOM signal to the local dynamic dielectric function of the sample. We model the tr‐SNOM signal using the point dipole model, accounting for the time‐dependent response of both the sample and tip. We first measure the tip response using a reference measurement on SiO_2_, a transparent dielectric. Accounting for the tip response, we then analyze the response of monolayer and multilayer WS_2_.

### 5.1 Active Tip Dynamics

As mentioned above, uncoated Si AFM tips show an increased pump–probe modulation compared to tips with a PtIr coating. Although the lack of a coating makes the tip more durable, withstanding longer optical alignment and higher fluences, the optical response of Si also adds to the NF enhancement. To assess this enhancement, we measure the tr‐SNOM signal on an SiO_2_ region of an AFM test grating using the same photon energies. Probing in the visible at 1.9 eV, a *pump‐off* SNOM signal is also expected, since the photon energy is above the bandgap of Si.

Figure 6 shows the amplitude and phase of the tr‐SNOM signal acquired in the location marked in the AFM inset. Since SiO_2_ is not excitable at the pump energy of 3.0 eV, no contribution to a pump–probe signal is expected, and signal dynamics are instead attributed to the AFM tip. The pump energy coincides with band nesting in the Si band structure, exciting electrons efficiently into the conduction band. The extra charge carriers lead to a dynamic contribution to the dielectric function of Si [31, 32]. We model the SNOM signal in Figure 6 with a single exponential in the dielectric function of the tip with a complex offset to account for longer lived dynamics.

(11) $$\varepsilon \left(t\right)=\varepsilon_{\text{static}}+\theta \left(t\right)\left(A\;e^{-t/\tau }+\varepsilon_{\text{offset}}\right),$$

where θ(t) is the Heaviside step function, $\varepsilon_{\text{static,tip}}=14.91+i0.12$ is the dielectric function of Si at 643 nm, and the parameters $A_{\text{tip}}$, $\tau_{\text{tip}}$, and $\varepsilon_{\text{offset,tip}}$ describe the dynamic response of the tip. The modified dielectric function of the tip is used in a point dipole model [16] to avoid additional assumptions on the spatial distribution and homogeneity of the excited charge carriers inside the tip. Using the finite dipole model [33] produces a similar result. The static dielectric function of SiO_2_ is taken to be $\varepsilon_{SiO_{2}}=2.12$ [34]. Fitting the time‐resolved data, shown as a solid line, determines the dynamic dielectric function of the tip, with the fit parameters collected in Table 1. The time constant was determined as $\tau_{\text{tip}}=\left(4.5\pm 3.2\right)$ ps, which is compatible with transient photoconductivity measured in Si nanostructures. The large relative uncertainty in determining $\tau_{\text{tip}}$ is due to the small amplitude of the tr‐SNOM signal, and the following small signal‐to‐noise ratio. Bergren et al. determined an initial decay of photoconductivity of ∼2 ps after pumping at 3 eV, attributed to cooling of hot carriers. A subsequent decay on ns timescales is ascribed to long‐lived excitons, both dynamics being dependent on the size of the nanostructures [35]. Tr‐SNOM measurement on Si showed similar kinetics with a slightly increased modulation in |Δσ/σ|. Parametrizing the dynamic dielectric function of the Si tip as an initial pump‐induced excitation, we are now able to disentangle the tip and sample contribution to the tr‐SNOM signal.

**FIGURE 6:**
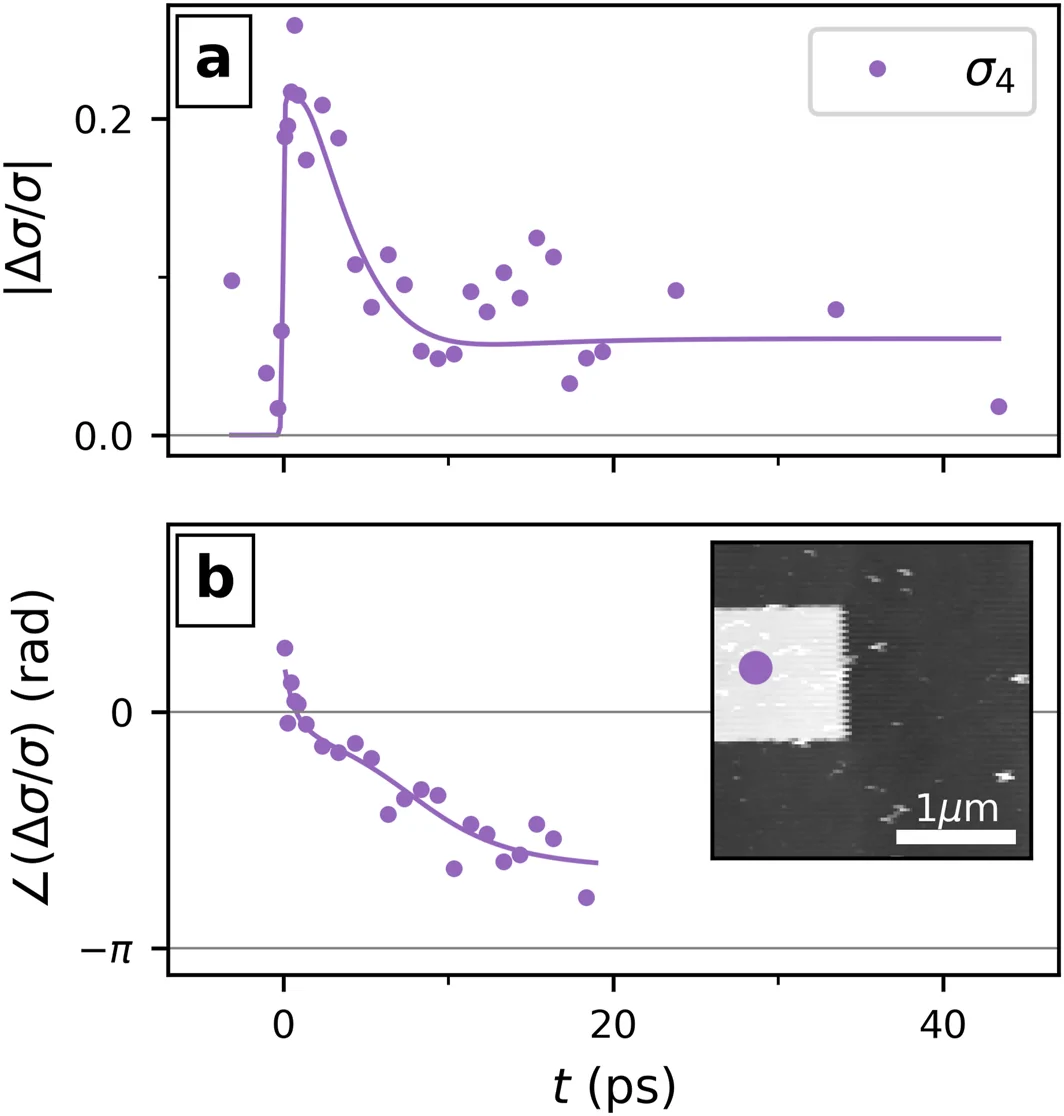
Time‐resolved SNOM signal on SiO_2_, acquired with an uncoated Si tip. The fit, shown as a solid line, considers a time‐dependent dielectric function of the tip, according to Equation (11). The AFM inset marks the acquisition site with a dot, in an SiO_2_ area on an AFM test grating (TGQ1, Tips‐Nano). The scan direction is alternating on the fast axis.

**TABLE 1:** Table of fit parameters determining the dynamic dielectric functions of the Si tip and WS_2_ sample, according to Equation (11).

Material	A	τ (ps)	$\varepsilon_{\text{offset}}$
Si tip	−10.6±1.8	4.5±3.2	(0.9+i2.0)±(2.1+i0.7)
WS_2_ monolayer	(5.5+i4.0)±(0.8+i0.8)	2.1±0.5	(−0.1−i0.2)±(0.1+i0.1)
WS_2_ multilayer	(0.08+i0.01)±(0.03+i0.03)	3.4±0.6	(0.08+i0.01)±(0.03+i0.03)

*Note:* The fits are shown in Figures 6 and 7. The dynamic dielectric functions are plotted in Figure 8.

### 5.2 Monolayer and Multilayer Dynamics

Using a similar model for the dielectric function of WS_2_, the tr‐SNOM signal provides a quantitative description of the ultrafast evolution of material properties at the nanoscale. Figure 7 shows NF kinetics $\left(\sigma_{4}\right)$, taken on a WS_2_ monolayer region ((a) and (c)) and a multilayer region ((b) and (d)). The kinetic traces are similar to the ones depicted in Figure 5b,d. The dataset plotted in Figure 7a,c is also plotted in the complex plane as black dots in Figure 1b. When modeling the tr‐SNOM signal, we use the transient dielectric function of WS_2_ as described by Equation (11). $\varepsilon_{\text{static}}$ at the probe energy of 1.9 eV, just below the A‐exciton resonance of 1.973 eV, is taken from literature [36]. The transient dielectric function of the AFM tip, as determined in the previous section, was fixed while calculating the SNOM signal with the point dipole model. The resulting fits are shown as solid lines in Figure 7, describing both monolayer and multilayer dynamics. The model provides a complex amplitude and offset and a decay rate of a dynamic contribution. Considering the phenomenological modeling approach, as well as ignoring NF contributions by the substrate, the fit results represent a qualitative characterization of the sample. Figure 8 shows the evolution of the local dielectric functions, together with the previously determined dielectric function of the tip. Both WS_2_ monolayer and multilayer show a pronounced photoinduced increase in $\varepsilon_{2}$. Although the monolayer also exhibits a distinct increase in $\varepsilon_{1}$, it is reduced on the multilayer. Both measurements show time constants on the order of few ps, with $\tau_{\text{monolayer}}=\left(2.1\pm 0.5\right)$ ps and $\tau_{\text{multilayer}}=\left(3.4\pm 0.6\right)$ ps. All fit parameters are collected in Table 1.

**FIGURE 7:**
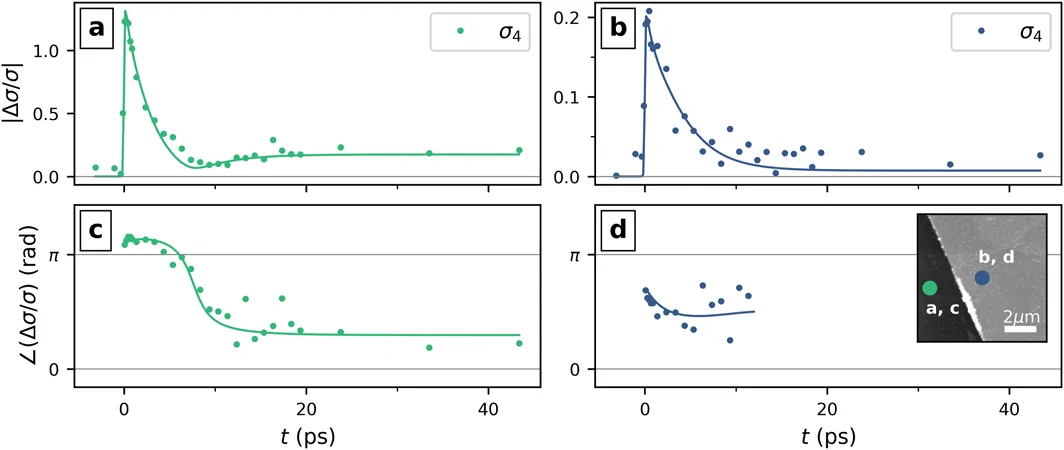
tr‐SNOM signal at different locations on WS_2_ close to a monolayer–multilayer boundary, marked in the AFM inset. (a) and (c) show the amplitude and phase of near‐field kinetics on monolayer WS_2_. (b) and (d) show different kinetic contributions on the multilayer side of the boundary. The near field was fitted, considering a time‐dependent dielectric function of the Si AFM tip, and the WS_2_ sample (Equation (11)). The optical phase in (d) is phase‐blanked where the signal amplitude falls below the noise level.

**FIGURE 8:**
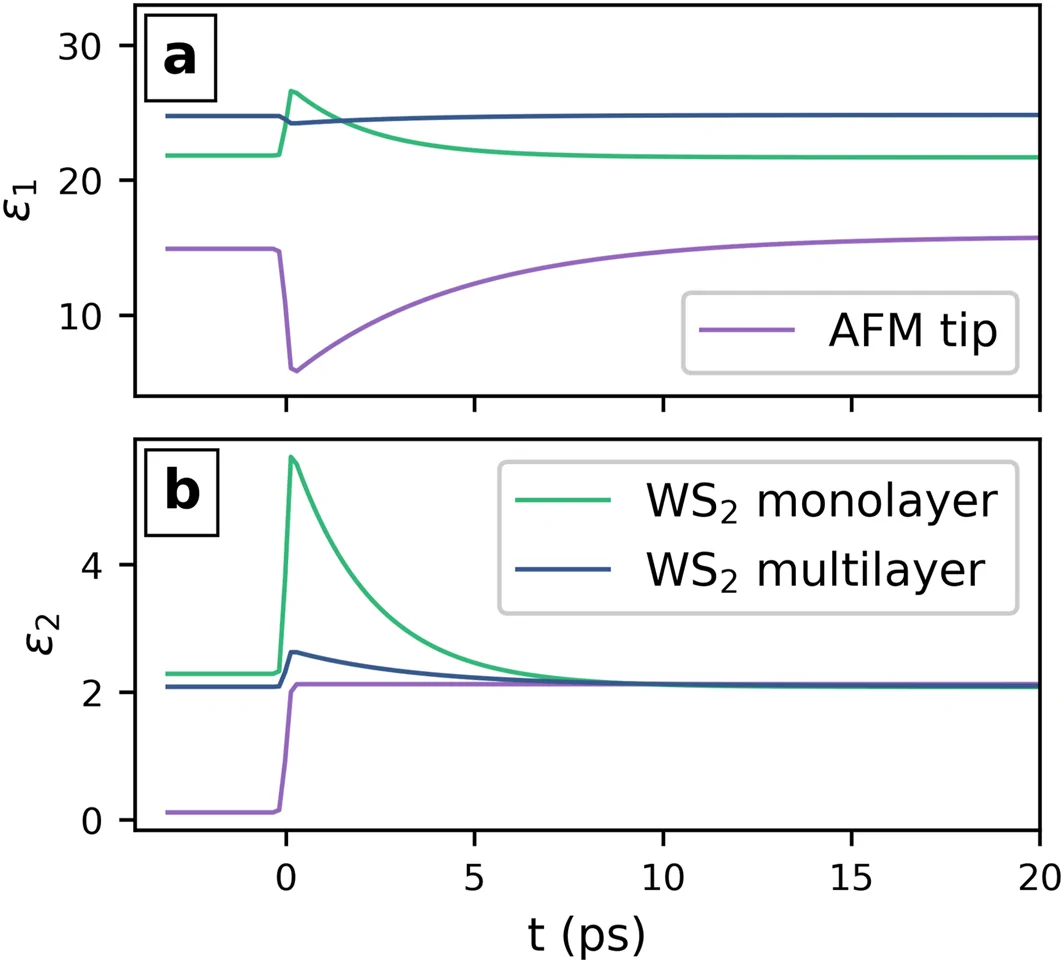
Modeled transient dielectric function of AFM tip (purple), WS_2_ monolayer (green), and WS_2_ multilayer (blue) after photo‐excitation at t=0. Real part $\varepsilon_{1}$ in (a), and imaginary part $\varepsilon_{2}$ in (b). See text for details of the models.

The dynamics are in agreement with femtosecond spectroscopy results on these materials [37, 38, 39, 40]. After above bandgap excitation, signal dynamics were found to be arising from peak shifts and amplitude reduction of the exciton response. These two phenomena cannot be disentangled from the current single‐color measurements. To avoid over‐determination of the model, we chose the phenomenological but general approach to directly model a dynamic dielectric function. Our results therefore demonstrate the isolation of different time‐resolved and localized contributions to the tr‐SNOM signal. By first isolating the tip response, we were able to determine the time‐resolved dielectric function of the sample.

## 6 Conclusion

We have defined a general tr‐SNOM signal Δσ/σ describing the time‐resolved NF response of the sample and presented an acquisition scheme using stroboscopic phase‐domain sampling. We demonstrated this method on the WS_2_ monolayer and multilayer, using a 200‐kHz repetition rate laser to pump above the bandgap at 3.0 eV and probe the A‐exciton response at 1.9 eV. The NF character was confirmed via signal–distance curves by identifying BG and NF contributions in signal kinetics and by resolving the localization of the dynamic NF at different positions in the monolayer–multilayer boundary region. We quantified the dynamic response of the AFM tip by measuring the tr‐SNOM signal on the transparent dielectric SiO_2_. Including the tip response in modeling the dynamic NF on WS_2_, we were able to describe the local time‐dependent dielectric function after the photo‐excitation of the monolayer and multilayer. We observed greatly enhanced pump‐modulation of the monolayer signal compared to the multilayer, with a faster decay. This marks the first realization of tr‐SNOM using sparse sampling in the time domain. The tr‐SNOM signal Δσ/σ is material‐specific, localized on the nanoscale, and able to resolve ultrafast processes.

## 7 Experimental Section

In the experiments, the pump photon energy is tuned to 3.0 eV (409 nm), whereas the probe energy is 1.9 eV (643 nm). The diameter of the pump beam in focus was estimated to be 4μm, which serves as a lower bound for the spot size of the pump beam, for which the alignment camera lacked sensitivity. The pump energy was measured at 200μW, leading to a pulse of ∼1nJ at 200 kHz and a peak fluence of $∼16\;mJ/{cm}^{2}$. The probe energy was measured at 50μW before the beam splitter, leading to ∼0.125nJ per pulse and a peak fluence of $∼2\;mJ/{cm}^{2}$.

The instrument response function has a width of σ=(77±1)fs, determined by fitting the onset of the far‐field pump–probe signal with an error function. The vibrating mirror modulates the optical path length of the reference arm at ∼300 Hz. For all pshet tr‐SNOM measurements in this paper, 800 000 samples were acquired (4 s acquisition time) for every presented data point. 1 200 000 samples (6 s) were acquired for the retraction curve presented in Figure 3 (pair of *pump‐on*, *pump‐off* data points), and 600 000 (3 s) for the shd tr‐SNOM data presented in the Supporting Information S1.

The AFM tips are commercially available Si tips (Arrow NC, Nanoworld) or coated Si tips (Arrow NCPt, Nanoworld), with the nominal resonance frequency of ∼275kHz with an amplitude of 58 nm in contact. For alignment and characterization of the AFM tip, we use a calibration grating (TGQ1, Tips‐Nano), featuring SiO_2_ squares on a Si substrate. Measurements on WS_2_ were performed on monolayer and multilayer regions on a Si substrate terminated by ∼300nm of SiO_2_, prepared by exfoliation [41].

## Author Contributions

**Philipp Schwendke:** conceptualization, methodology, software, data curation, investigation, validation, formal analysis, visualization, writing – original draft, writing – review and editing. **Julia Stähler:** conceptualization, supervision, funding acquisition, writing – review and editing, project administration. **Samuel Palato:** conceptualization, methodology, project administration, writing – review and editing, supervision, formal analysis.

## Supporting information


Supporting Information S1

## Data Availability

The data that support the findings of this study are available from the corresponding author upon reasonable request.

## References

[1] G. Bar , R. Brandsch , M. Bruch , L. Delineau , and M.‐H. Whangbo Surface Science 444 (2000): 1–3.

[2] M. Munz , J. Poon , W. Frandsen , B. R. Cuenya , and C. S. Kley , “Nanoscale Electron Transfer Variations at Electrocatalyst–Electrolyte Interfaces Resolved by *in Situ* Conductive Atomic Force Microscopy,” Journal of the American Chemical Society 145, no. 9 (2023): 5242–5251, 10.1021/jacs.2c12617. PMC9999420 36812448

[3] A. Shiotari , J. Nishida , A. Hammud , et al. Science Advances 11 (2025): 24. 10.1126/sciadv.adu1415 PMC12154227 40498825

[4] N. Ansari , E. Mohebbi , and F. Gholami Journal of Applied Physics 127 (2020): 6.

[5] M. Rothe , Y. Zhao , J. Müller , et al., “Self‐Assembly of Plasmonic Nanoantenna–Waveguide Structures for Subdiffractional Chiral Sensing,” ACS Nano 15, no. 1 (2020): 351–361, 10.1021/acsnano.0c05240. 33233888

[6] R. Verre , D. G. Baranov , B. Munkhbat , J. Cuadra , M. Käll , and T. Shegai , “Transition Metal Dichalcogenide Nanodisks as High‐Index Dielectric Mie Nanoresonators,” Nature Nanotechnology 14, no. 7 (2019): 679–683, 10.1038/s41565-019-0442-x. 31061517

[7] M. M. Wiecha , S. Al‐Daffaie , A. Bogdanov , et al., “Direct Near‐Field Observation of Surface Plasmon Polaritons on Silver Nanowires,” ACS Omega 4, no. 26 (2019): 21962–21966, 10.1021/acsomega.9b03036. PMC6933792 31891075

[8] R. Hillenbrand , Y. Abate , M. Liu , X. Chen , and D. N. Basov , “Visible‐to‐THz Near‐Field Nanoscopy,” Nature Reviews Materials 10, no. 4 (2025): 285–310, 10.1038/s41578-024-00761-3.

[9] M. Eisele , T. L. Cocker , M. A. Huber , et al., “Ultrafast Multi‐Terahertz nano‐Spectroscopy With Sub‐Cycle Temporal Resolution,” Nature Photonics 8, no. 11 (2014): 841–845, 10.1038/nphoton.2014.225.

[10] A. Pizzuto , E. Castro‐Camus , W. Wilson , W. Choi , X. Li , and D. M. Mittleman , “Nonlocal Time‐Resolved Terahertz Spectroscopy in the Near Field,” ACS Photonics 8, no. 10 (2021): 2904–2911, 10.1021/acsphotonics.1c01367.

[11] S. A. Dönges , O. Khatib , B. T. O’Callahan , et al., “Ultrafast Nanoimaging of the Photoinduced Phase Transition Dynamics in VO_2_ ,” Nano Letters 16, no. 5 (2016): 3029–3035, 10.1021/acs.nanolett.5b05313. 27096877

[12] A. J. Sternbach , F. L. Ruta , Y. Shi , et al., “Nanotextured Dynamics of a Light‐Induced Phase Transition in VO_2_ ,” Nano Letters 21, no. 21 (2021): 9052–9060, 10.1021/acs.nanolett.1c02638. 34724612

[13] K. Nishikawa , J. Nishida , M. Yoshimura , K. Nakamoto , T. Kumagai , and Y. Watanabe , “Metastability in the Insulator–Metal Transition for Individual Vanadium Dioxide Nanoparticles,” Journal of Physical Chemistry C 127, no. 33 (2023): 16485–16495, 10.1021/acs.jpcc.3c02151.

[14] M. Mrejen , L. Yadgarov , A. Levanon , and H. Suchowski , “Transient exciton‐polariton Dynamics in WSe _2_ by Ultrafast near‐field Imaging,” Science Advances 5, no. 2 (2019): eaat9618, 10.1126/sciadv.aat9618. PMC6358311 30746484

[15] Y. Wang , J. Nishida , K. Nakamoto , et al., “Ultrafast Nano‐Imaging of Spatially Modulated Many‐Body Dynamics in CVD‐Grown Monolayer WS_2_ ,” ACS Photonics 12, no. 1 (2024): 207–218, 10.1021/acsphotonics.4c01545.

[16] B. Knoll and F. Keilmann Optics Communications 182 (2000): 4–6.

[17] H. J. Woo , S. Lee , H. Kim , S. Jung , E. S. Lee , and J. Jahng , “Characterizing Nanoscale Spatiotemporal Defects of Multi‐Layered MoSe_2_ in Hyper‐Temporal Transient Nanoscopy,” Nanophotonics 14, no. 15 (2025): 2683–2693, 10.1515/nanoph-2025-0163. PMC12322730 40771419

[18] A. J. Sternbach , J. Hinton , T. Slusar , et al., “Artifact Free Time Resolved Near‐Field Spectroscopy,” Optics Express 25, no. 23 (2017): 28589, 10.1364/oe.25.028589.

[19] H. Wang , L. Wang , and X. G. Xu Nature Communications 7 (2016): 1.

[20] N. Ocelic , A. Huber , and R. Hillenbrand , “Pseudoheterodyne Detection for Background‐Free Near‐Field Spectroscopy,” Applied Physics Letters 89, no. 10 (2006): 101124, 10.1063/1.2348781.

[21] S. Palato , P. Schwendke , N. B. Grosse , and J. Stähler , “Pseudoheterodyne Near‐Field Imaging at kHz Repetition Rates via Quadrature‐Assisted Discrete Demodulation,” Applied Physics Letters 120, no. 13 (2022): 131601, 10.1063/5.0087187.

[22] G. Wurtz , R. Bachelot , and P. Royer , “Imaging a GaAlAs Laser Diode in Operation Using Apertureless Scanning Near‐Field Optical Microscopy,” European Physical Journal Applied Physics 5, no. 3 (1999): 269–275, 10.1051/epjap:1999139.

[23] M. Labardi , S. Patanè , and M. Allegrini , “Artifact‐Free Near‐Field Optical Imaging by Apertureless Microscopy,” Applied Physics Letters 77, no. 5 (2000): 621–623, 10.1063/1.127064.

[24] S. Mukamel , Principles of Nonlinear Optical Spectroscopy (Oxford Series on Optical and Imaging Sciences) (Oxford University Press, 1999).

[25] P. Schwendke , “Codeberg—Phips/SNOMpad,”2025, https://codeberg.org/phips/SNOMpad/src/branch/trSNOM‐publication.

[26] M. Specht , J. D. Pedarnig , W. M. Heckl , and T. W. Hänsch , “Scanning Plasmon Near‐Field Microscope,” Physical Review Letters 68, no. 4 (1992): 476–479, 10.1103/physrevlett.68.476. 10045906

[27] Y. Inouye and S. Kawata , “Near‐Field Scanning Optical Microscope With a Metallic Probe Tip,” Optics Letters 19, no. 3 (1994): 159, 10.1364/ol.19.000159. 19829577

[28] R. Hillenbrand and F. Keilmann , “Complex Optical Constants on a Subwavelength Scale,” Physical Review Letters 85, no. 14 (2000): 3029–3032, 10.1103/physrevlett.85.3029. 11005995

[29] M. B. Raschke and C. Lienau , “Apertureless Near‐Field Optical Microscopy: Tip–Sample Coupling in Elastic Light Scattering,” Applied Physics Letters 83, no. 24 (2003): 5089–5091, 10.1063/1.1632023.

[30] R. Trebino , Frequency‐Resolved Optical Gating: The Measurement of Ultrashort Laser Pulses (Springer US, 2000).

[31] M. A. Green , “Self‐Consistent Optical Parameters of Intrinsic Silicon at 300K Including Temperature Coefficients,” Solar Energy Materials and Solar Cells 92, no. 11 (2008): 1305–1310, 10.1016/j.solmat.2008.06.009.

[32] M. Combescot and J. Bok Journal of Luminescence 30 (1985): 1–4.

[33] A. Cvitkovic , N. Ocelic , and R. Hillenbrand , “Analytical Model for Quantitative Prediction of Material Contrasts in Scattering‐Type Near‐Field Optical Microscopy,” Optics Express 15, no. 14 (2007): 8550, 10.1364/oe.15.008550. 19547189

[34] I. H. Malitson , “Interspecimen Comparison of the Refractive Index of Fused Silica*,†,” Journal of the Optical Society of America 55, no. 10 (1965): 1205, 10.1364/josa.55.001205.

[35] M. R. Bergren , C. E. Kendrick , N. R. Neale , et al., “Ultrafast Electrical Measurements of Isolated Silicon Nanowires and Nanocrystals,” Journal of Physical Chemistry Letters 5, no. 12 (2014): 2050–2057, 10.1021/jz500863a. 26270492

[36] S. Calati , Q. Li , X. Zhu , and J. Stähler Physical Chemistry Chemical Physics (2021). 10.1039/d1cp03437e 34596640

[37] B. Tanda Bonkano , S. Palato , J. Krumland , et al. Physica Status Solidi 221 (2023): 1.

[38] C. E. P. Villegas , E. Marinho , P. Venezuela , and A. R. Rocha , “Optical Spectra and Exciton Radiative Lifetimes in Bulk Transition Metal Dichalcogenides,” Physical Chemistry Chemical Physics 26, no. 17 (2024): 13251–13260, 10.1039/d3cp05949a. 38634830

[39] M. Palummo , M. Bernardi , and J. C. Grossman , “Exciton Radiative Lifetimes in Two‐Dimensional Transition Metal Dichalcogenides,” Nano Letters 15, no. 5 (2015): 2794–2800, 10.1021/nl503799t. 25798735

[40] S. Calati , Q. Li , X. Zhu , and J. Stähler , “Dynamic Screening of Quasiparticles in WS2 Monolayers,” Physical Review B 107, no. 11 (2023): 115404, 10.1103/physrevb.107.115404.

[41] S. B. Desai , S. R. Madhvapathy , M. Amani , et al., “Gold‐Mediated Exfoliation of Ultralarge Optoelectronically‐Perfect Monolayers,” Advanced Materials 28, no. 21 (2016): 4053–4058, 10.1002/adma.201506171. 27007751

