# Supplementary material for: Time‐Resolved SNOM via Phase‐Domain Sampling
Source: Nanophotonics. 2026 Jun 22;15(12):e70178. doi: 10.1002/nap2.70178 (PMC13642175; doi:10.1002/nap2.70178)
Supplement: Supplementary file 1 [file NAP2-15-e70178-s001.pdf]

Supporting Information for: Time-resolved SNOM via phase-domain sampling

Philipp Schwendke, Julia Stähler, Samuel Palato
samuel.palato@hu-berlin.de

Contents

S1 UV/Vis and TA spectra of WS_2 **1**

S2 Self-homodyne modulation **2**

S3 SNOM signal modeling **3**

S4 Signal contribution of Si AFM tip **5**

S5 Pump-probe signal distance curves with Si tips **6**

S6 Pump-probe signal distance curves with PtIr tips **7**

S7 Comparison of tip-only signal on SiO_2/Si and fused silica **9**

S1 UV/Vis and TA spectra of WS_2

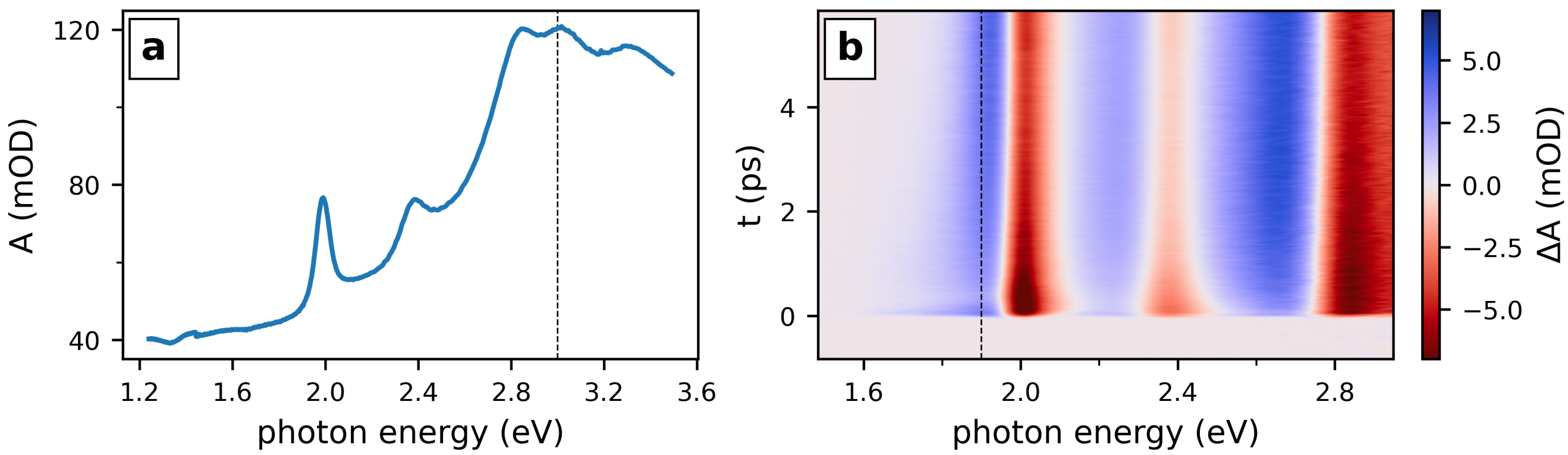

Figure S1: UV/Vis and TA spectra with pump and probe energies marked. (a): UV/Vis spectrum WS_2 monolayer on silica. The pump energy during tr-SNOM measurements of 3.0 eV is marked by a dashed line. (b): TA spectrum of the same sample showing signal kinetics during the first 5 ps. The probe energy during tr-SNOM measurements of 1.9 eV is marked by a dashed line.

Figure S1 shows absorbance spectra of WS_2 on silica. The static spectrum in (a) was taken in a UV/Vis spectrometer. A and B-exciton at 2.0 eV and 2.4 eV respectively a visible as peaks below the interband transition. The pump energy of 3.0 eV during tr-SNOM measurements is marked by a dashed line. Above bandgap excitation coincides with high absorbance of ~ 120 mOD.

The transient absorption (TA) spectrum in (b) displays the change in absorbance after excitation at 3.0 eV during the first 5 ps. In addition to a bleaching of the exciton transitions, the increase in optical density implies peakshifts after photo-excitation. At the probe energy of 1.9 eV, used during tr-SNOM measurements, a red shift of the A-exciton increases the optical density.

S2 Self-homodyne modulation

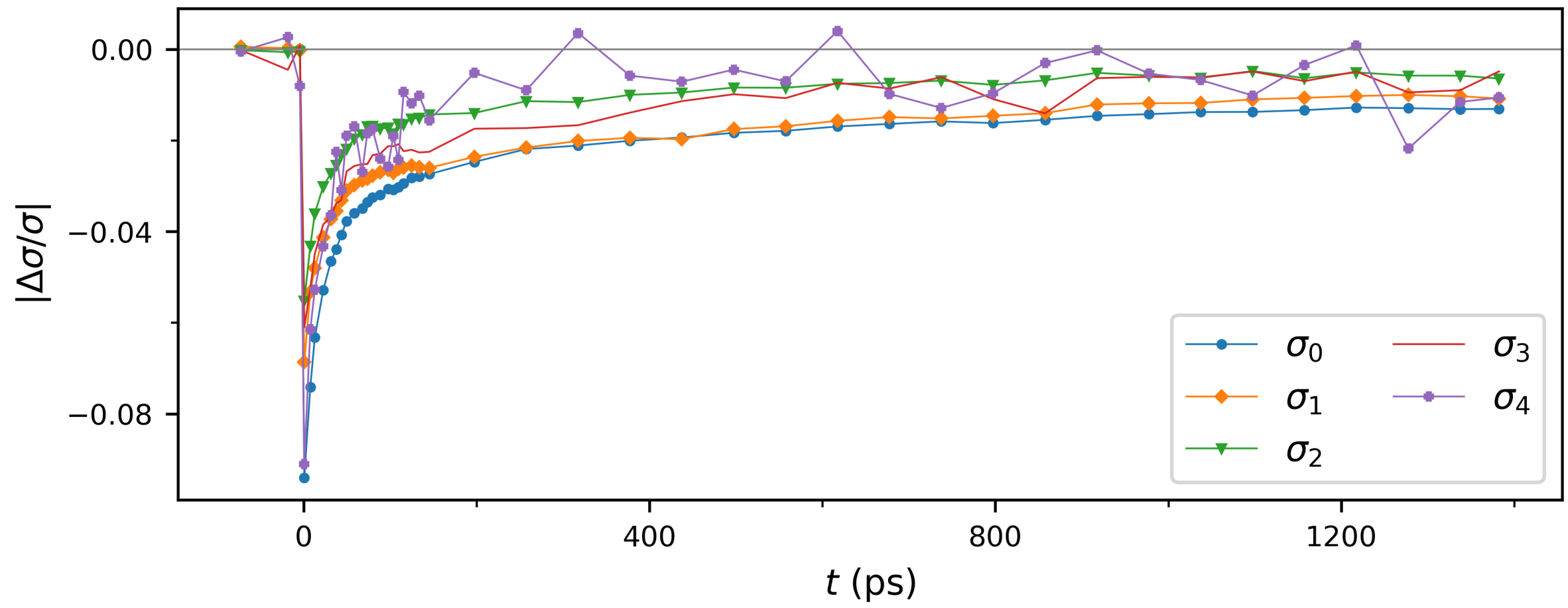

Figure S2: Time-resolved SNOM signal for different harmonic orders, using self-homodyne modulation.

During pshet measurements, the phasor of the electric field detected by the photo diode E_{sig} consists of the scattered fields E_{NF} and E_{BG}, as well as the reference arm E_{ref}. Only E_{NF} and E_{BG} are modulated by photo-excitation.

$$|E_{\text{sig,on}}(t)|^2 = |E_{\text{sig,off}} + \Delta E_{\text{NF}}(t) + \Delta E_{\text{BG}}(t)|^2 \tag{S1}$$

$$\begin{aligned}|E_{\text{sig,on}}(t)|^2 = {} & |E_{\text{sig,off}}|^2 + |\Delta E_{\text{NF}}(t)|^2 + |\Delta E_{\text{BG}}(t)|^2 + \Delta E_{\text{NF}}(t)\Delta E^*_{\text{BG}}(t) \\ & + E_{\text{sca}}\Delta E^*_{\text{NF}}(t) + E_{\text{ref}}\Delta E^*_{\text{NF}}(t) + E_{\text{sca}}\Delta E^*_{\text{BG}}(t) + E_{\text{ref}}\Delta E^*_{\text{BG}}(t) + c.c.\end{aligned} \tag{S2}$$

During self-homodyne (shd) measurements, $E_{\text{ref}} = 0$. Using Fourier decomposition

$$E_{\text{NF/BG}} = \sum_n \sigma_{n,\text{NF/BG}} e^{in(\theta_{\text{tap}} - \theta_C)} \tag{S3}$$

the photodiode then detects the signal

$$\begin{aligned}|E_{\text{sig,on}}(t)|^2 = \sum_n \sum_m [& \sigma_{n,\text{NF}}\sigma^*_{m,\text{NF}} + \sigma_{n,\text{BG}}\sigma^*_{m,\text{BG}} + \Delta\sigma_{n,\text{NF}}\Delta\sigma^*_{m,\text{NF}} + \Delta\sigma_{n,\text{BG}}\Delta\sigma^*_{m,\text{BG}} \\ & + (\sigma_{n,\text{NF}}\sigma^*_{m,\text{BG}} + \Delta\sigma_{n,\text{NF}}\Delta\sigma^*_{m,\text{BG}} + \sigma_{n,\text{NF}}\Delta\sigma^*_{m,\text{NF}} + \sigma_{n,\text{BG}}\Delta\sigma^*_{m,\text{NF}} \\ & + \sigma_{n,\text{NF}}\Delta\sigma^*_{m,\text{BG}} + \sigma_{n,\text{BG}}\Delta\sigma^*_{m,\text{BG}} + c.c.)]\, e^{i(n-m)(\theta_{\text{tap}} - \theta_C)}\end{aligned} \tag{S4}$$

In this double sum, higher order of BG contribution will become small and negligible. However, multiplication with the 0-order BG will always dominate the sums as $|\sigma_0| \gg |\sigma_{m>0}|$. We can ascribe the higher harmonics of the photodiode signal to the remaining terms in the double sum above.

$$|E_{\text{sig}}(t)|^2 = \sum_n \tau'_n(t) e^{in(\theta_{\text{tap}} - \theta_C)} \tag{S5}$$

$$\tau_{n \gg 0}(t) \approx \sigma_{n,\text{NF}}\sigma^*_{0,\text{BG}} + (\Delta\sigma_{n,\text{NF}}(t) + \sigma_{n,\text{NF}})\Delta\sigma^*_{0,\text{BG}}(t) + c.c. \tag{S6}$$

In this term, the time-dependent near-field is only accessible as a product with the time-dependent background. Furthermore, the time-dependent BG will always dominate since in general $\Delta\sigma_{n,\text{NF}}(t) \ll \sigma_{n,\text{NF}}$. This is illustrated by time-resolved shd measurements shown in Figure S2. All signal harmonics show similar dynamics as well as similar pump-probe modulation depth as expected. The measurement was done using a PtIr coated AFM tip, on a WS_2 multilayer, 3.0 eV (415 nm) pump energy and 1.9 eV (642 nm) probe energy.

S3 SNOM signal modeling

To model the near-field (NF) enhancement, we describe the NF contribution of the scattered light by the scattering coefficient σ_{NF}.

$$E_{\mathrm{NF}} \propto p_{\mathrm{eff}} = \sigma_{\mathrm{NF}} E_0 \tag{S7}$$

$$\sigma_{\mathrm{NF}} = (1 + cr)^2 \alpha_{\mathrm{eff}} \tag{S8}$$

The description contains the Fresnel reflection for p-polarized light r, which itself depends on the dielectric function of the area around the scattering site, a factor c which scales the amount of indirect illumination of the AFM tip[1], and finally the effective polarizability of the tip-sample junction α_{eff}. To describe the polarizability, we use the point dipole model.

$$\alpha_{\mathrm{eff}} = \frac{\alpha_{\mathrm{tip}}}{1 - \frac{\alpha_{\mathrm{tip}}\beta}{16\pi(h+a)^3}} \tag{S9}$$

Equation (S9) accounts for indirect illumination as described in Equation (S8)[2, 3]. Equation (S9) describes the sample response via the formation of image charges at a boundary of two dielectrics, scaled by β[4].

$$\beta = \frac{\varepsilon_s - \varepsilon_m}{\varepsilon_s + \varepsilon_m} \tag{S10}$$

ε_s is the dielectric function of the sample surface and ε_m is the dielectric function of the the medium in between, in our case air. Furthermore, Equation (S9) describes the response of the AFM tip as a conducting sphere with radius $a \ll \lambda$ and isotropic polarizability α_{tip}[5, 6].

$$\alpha_{\mathrm{tip}} = 4\pi\epsilon_{\mathrm{m}}\epsilon_0 a^3 \frac{\epsilon_{\mathrm{t}} - \epsilon_{\mathrm{m}}}{\epsilon_{\mathrm{t}} + 2\epsilon_{\mathrm{m}}} \tag{S11}$$

Here ε_t is the dielectric function of the tip. To model tapping modulation, we vary the tip height h in Equation (S9).

$$h(\theta_{\mathrm{tap}}) = A(1 + cos(\theta_{\mathrm{tap}})) \tag{S12}$$

A is the tapping amplitude. We then compute the NF scattering as described above. The spectrum of $\sigma_{\mathrm{NF}}(\theta_{\mathrm{tap}})$ then returns the complex signal harmonics σ_n.

There are of course limitations to this approach. As mentioned in the main text, one could use a more sophisticated physical model of NF enhancement e.g. the finite dipole model. The point dipole model was then chosen because of its conceptual simplicity, allowing fewer fit parameters and less assumptions about the spatial distribution of excitations in both sample and AFM tip.

Second, it should be mentioned that this model is a bulk material approach, not taking into account reflection or NF enhancement by a possibly layered substrate. The description of layered samples is described for the point dipole[7] and the finite dipole model[8]. We believe our modeling of the sample, indeed using a bulk material approach, leads to qualitatively reasonable results. A major correction of a treatment as a multilayered system is the inclusion of reflections off of the substrate surface. These are also included in the reflection coefficient c. Furthermore, we justify neglecting a substrate contribution to the detected NF enhancement onsidering the lack of signal dynamics of SiO2 in the visible spectrum. This means, the substrate may very well scale the SNOM signal, but we expect the signal kinetics to be unaltered.

References

[1] L. Mester, A. A. Govyadinov, R. Hillenbrand, *Nanophotonics* **2021**, *11*, 2 377.

[2] M. B. Raschke, C. Lienau, *Applied Physics Letters* **2003**, *83*, 24 5089.

[3] A. Cvitkovic, N. Ocelic, R. Hillenbrand, *Optics Express* **2007**, *15*, 14 8550.

[4] J. D. Jackson, *Classical Electrodynamics*, John Wiley & Sons Inc, third edition, **1998**.

[5] C. Bohren, *Absorption and scattering of light by small particles*, Wiley, New York, **1983**.

[6] M. Fox, *Optical Properties of Solids*, Oxford University Press, second edition, **2010**.

[7] J. Aizpurua, T. Taubner, F. J. García de Abajo, M. Brehm, R. Hillenbrand, *Optics Express* **2008**, *16*, 3 1529.

[8] B. Hauer, A. P. Engelhardt, T. Taubner, *Optics Express* **2012**, *20*, 12 13173.

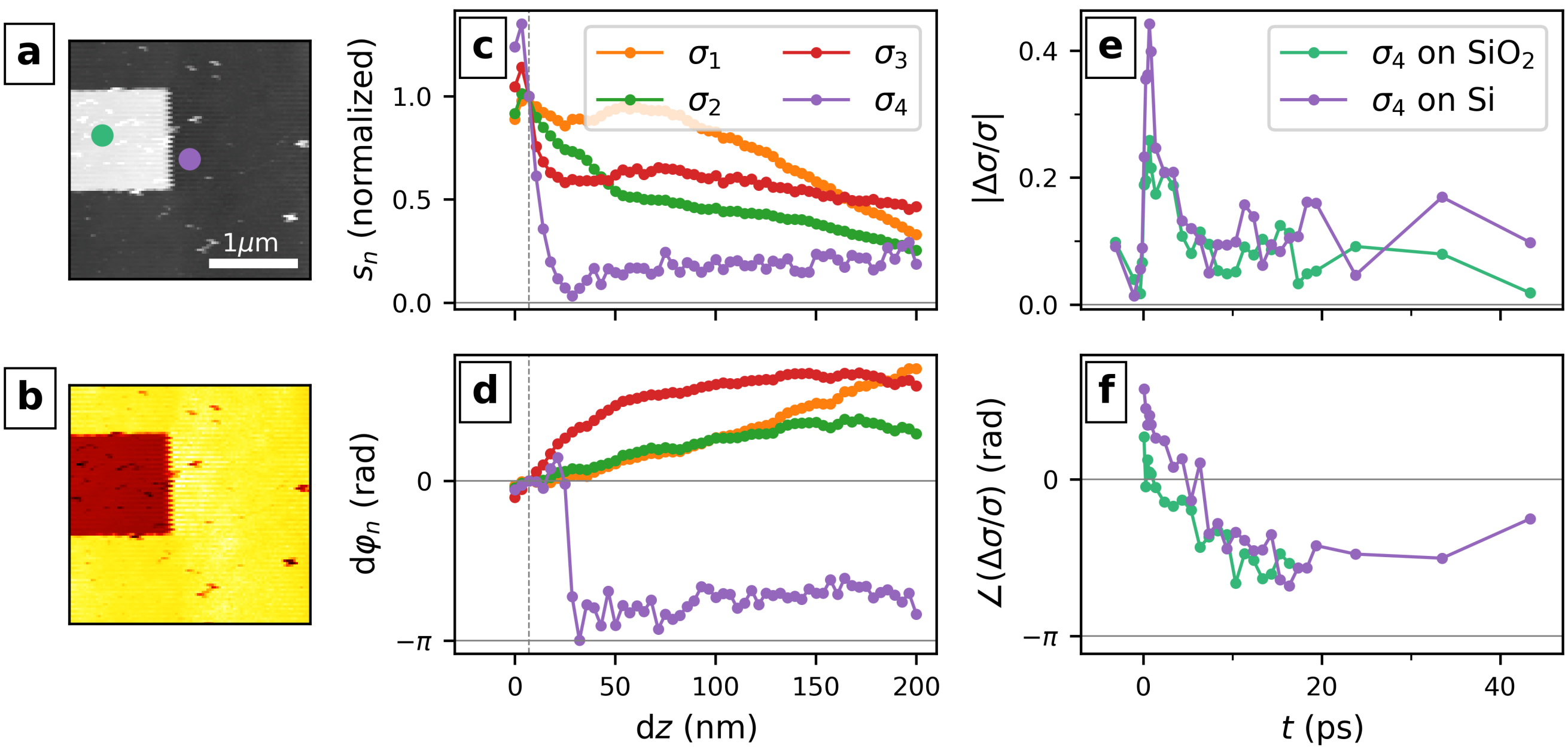

Figure S3: SNOM measurements of AFM calibration grating TGQ1, featuring SiO_2 squares on a Si substrate. (a): AFM z-image showing both *trace* and *retrace* directions in one image. The acquisition sites in (e) and (f) are marked by colored circles. (b): Pshet SNOM image of of the same measurement as (a), taken with an uncoated Si AFM tip and CW HeNe laser at 1.96 eV photon energy. Shown is the amplitude of the 4th harmonic s_4 (c) and (d) show SNOM signal amplitude and phase respectively of a retraction curve on Si (purple circle in (a)). The signal was acquired with a pulsed laser at 1.9 eV photon energy. The distance with maximum tapping amplitude, from where the AFM tip loses contact to the sample, is marked by a vertical dashed line. (e) and (f) show tr-SNOM signal amplitude and phase respectively of σ_4, acquired in the marked locations in (a).

S4 Signal contribution of Si AFM tip

Figure S3 shows SNOM measurement on Si and SiO_2, with the aim of characterizing a NF response of uncoated Si AFM tips. The retraction curve shown in (c) and (d) was taken with a pulsed laser with 1.9 eV photon energy on Si, the location marked in purple in (a). σ_4, while still containing a small complex offset, seems to be dominated by NF while in contact. Adding pump modulation with 3.0 eV photon energy, (e) and (f) show SNOM signal kinetics on Si in purple. The tr-SNOM signal acquired on SiO_2 is plotted in green and shows similar kinetics.

We can conclude that the kinetics shown in (e) and (f) are a material response by Si. The comparable signal amplitude in (e), as well as the NF character of the probe pulse shown in (c) and (d), further indicate that a considerable amount of that signal is a response by the Si AFM tip. Hence, the dynamic dielectric function of the AFM tip, obtained by modeling the response on SiO_2, plottet in green in (e) and (f), is used to model tr-SNOM measurements presented in the main text.

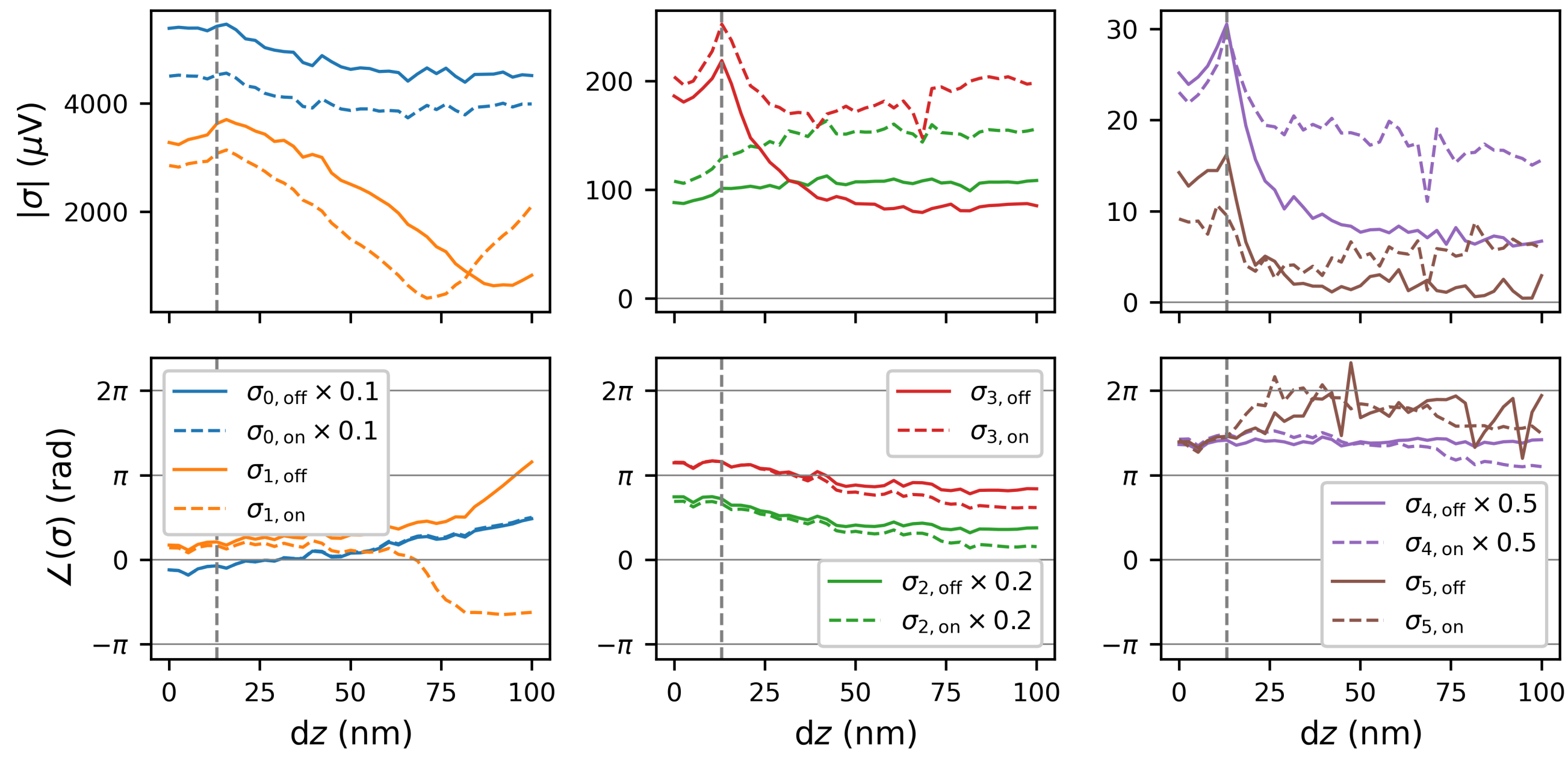

Figure S4: SNOM signal-distance curves for different harmonic orders, each showing *pump-off* (solid) and *pump-on* (dashed) signals. Acquired with uncoated SI AFM tips (Arrow NC, NanoWorld). This is the full data set presented in Figure 3 in main text, without subtraction of a complex offset signal.

S5 Pump-probe signal distance curves with Si tips

Figure S4 shows harmonics 0 - 5 of SNOM signal-distance curves, acquired on WS_2 with a Si AFM tip. The figure shows the full data set presented in Figure 3 in the main text. Here, many contribution are visible. A near-field contribution should decay within $\sim 10\,\mathrm{nm}$, leading to a low signal amplitude and noisy phase (like $\sigma_{5,\mathrm{off}}$ after 40 nm). All other contributions are considered background.

The optical phase relative to the retracted distance dz also indicates the signal origin, as a shifting phase suggests a reflection on the sample surface. In the case of a background signal, a reflection off of tip shaft and sample surface always shows a shifting phase. For the near-field, indirect illumination is affected by reflections off of the sample surface, and can lead to a pronounced phase dependency if c in $(1 + cr)^2\alpha_{\mathrm{eff}}$ is very large. Background and indirect illumination do not necessarily need to shift in the same direction with increasing dz.

We also observe signals converging towards a complex value > 0 (complex offset), e.g. $\sigma_{3,4,\mathrm{off}}$. We attribute this modulation without sample interaction to scattering off of the AFM tip (tip-only signal), modulated by the tip movement inside the focused laser spot. In the complex plane, this offset can easily be identified and subtracted.

Figure S4 shows a background contribution in all harmonics. $\sigma_{3,4,5}$ show near-field character with signal amplitude dropping quickly after loosing tip-sample contact. However, in σ_3 and σ_4, a defined phase off-contact indicates a background signal.

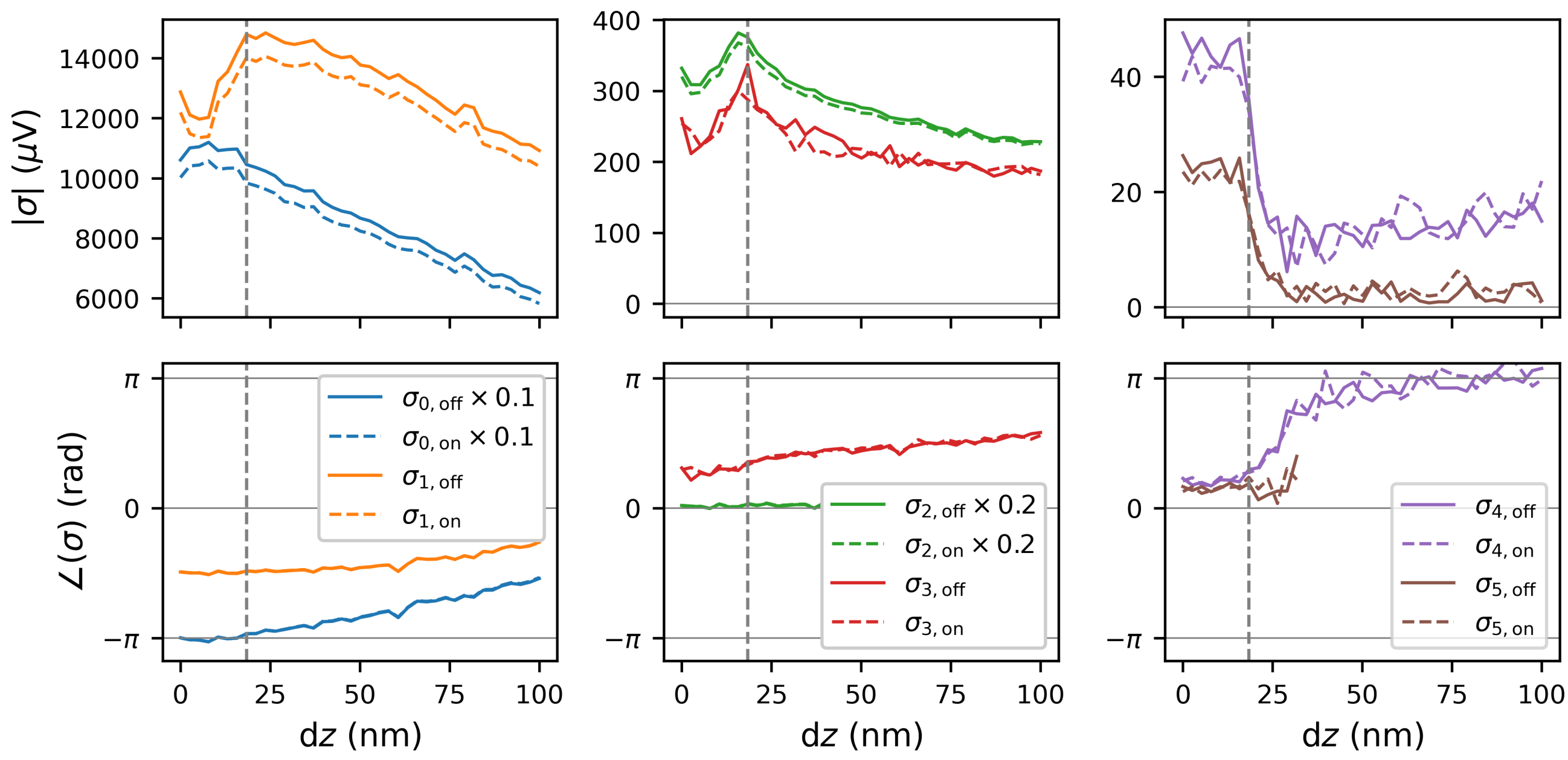

Figure S5: SNOM signal-distance curves for different harmonic orders, each showing pump-off (solid) and pump-on (dashed) signals. Acquired with PtIr coated AFM tips (Arrow NCPt, NanoWorld) on multilayer WS_2.

S6 Pump-probe signal distance curves with PtIr tips

Figure S5 shows signal-distance curves on WS_2 acquired with PtIr coated AFM tips, using pseudo-heterodyne modulation. The pump energy is 3.0 eV (412 nm), and the probe energy 1.9 eV (648 nm). The pump-probe delay is 0.5 ps. The dotted line marks the z position with the maximal AFM tapping amplitude (snap-off).

σ_2 and σ_3 are dominated by background scattering, which is still present in σ_4, at larger tip-sample distances. σ_4 and σ_5 show a pump-probe contrast while in-contact that decays with decaying near-field signal. The decay of σ_5 beneath the noise floor for d$z > 30$ nm supports the identification as NF. Furthermore the pump-modulation of $\sigma_{4,5}$ also vanishes for d$z > 30$ nm. The difference of $\sigma_{4,5}$ *pump-on* and *pump-off* signals, while in contact (d$z < 20$ nm), is the time-resolved NF signal $\Delta\sigma_n$ mentioned in the main text.

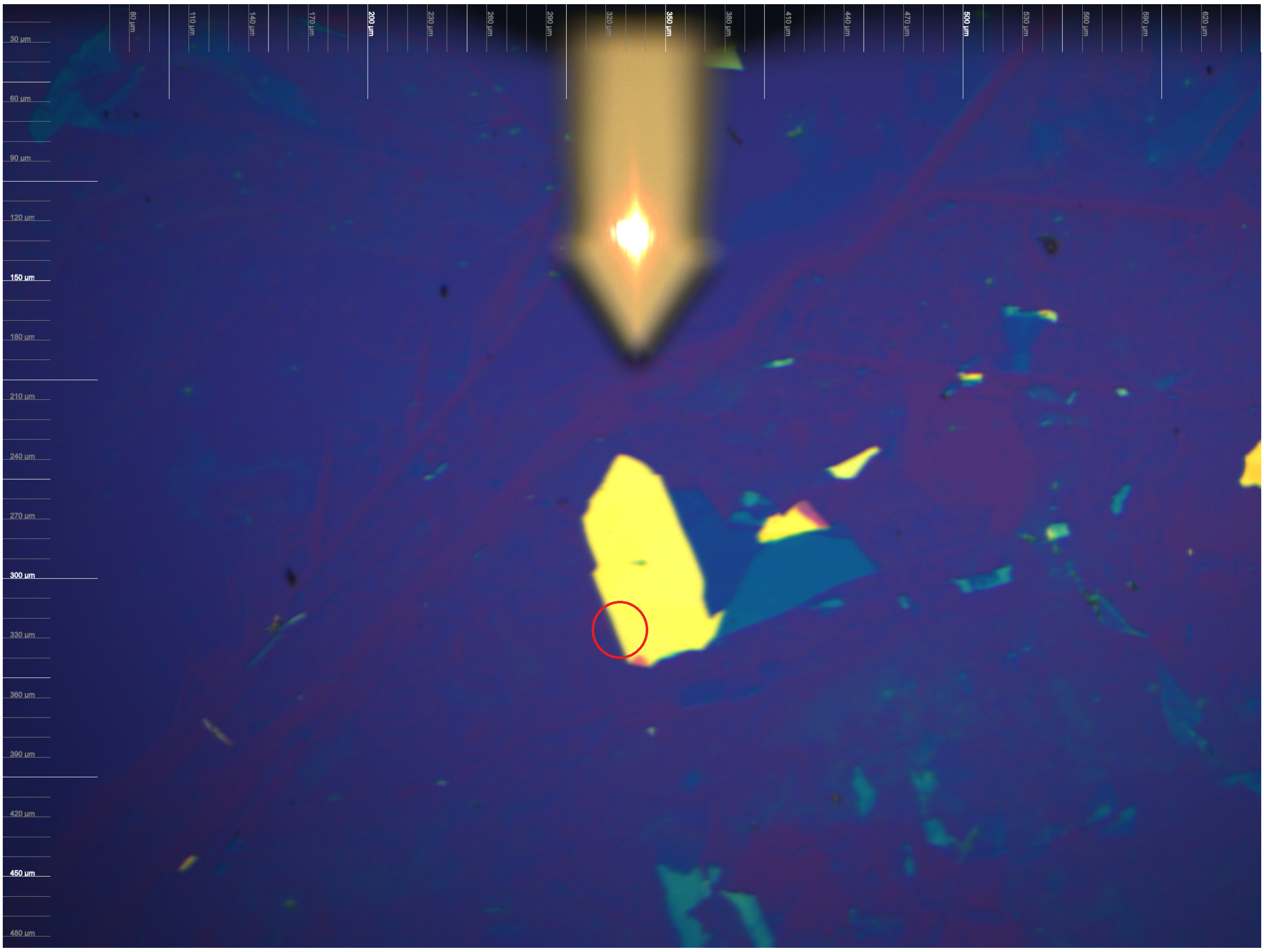

Figure S6: Optical image of exfoliated WS_2 on SiO_2/Si sample. The red circle marks the acquisition sites where the AFM images in the main text were taken. The substrate appears purple, while the the monolayer regions appear blue. The multilayer island shows a yellow contrast.

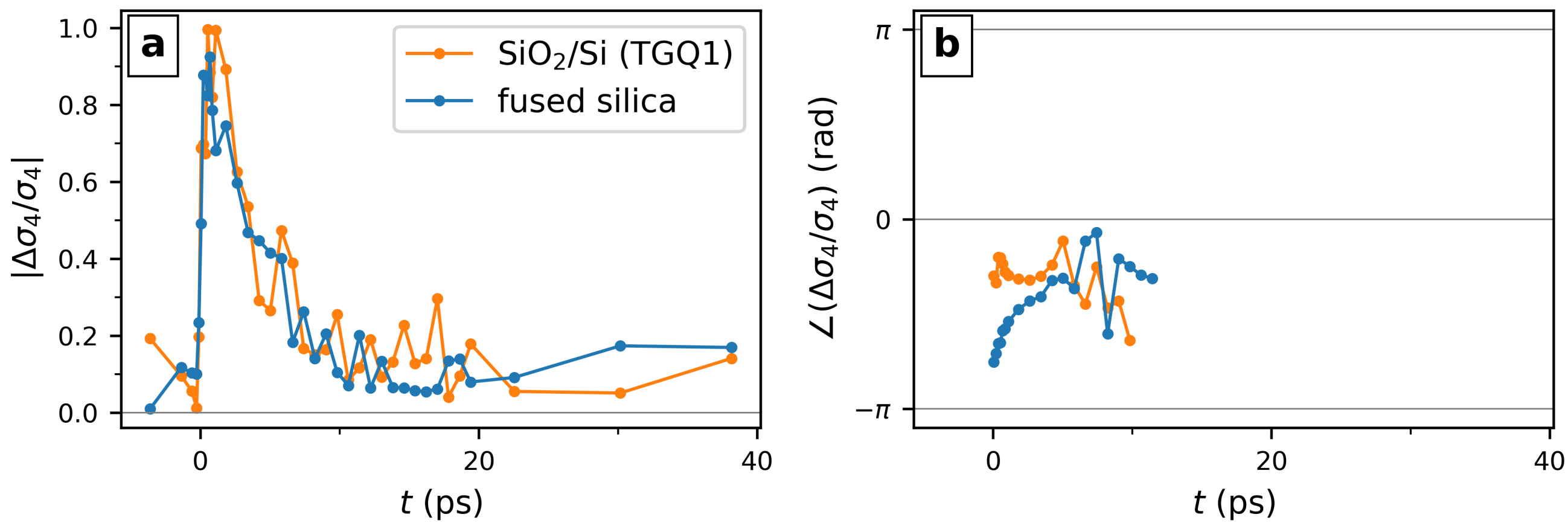

Figure S7: Tr-SNOM signal kinetics acquired on SiO_2/Si, and on fused silica, with uncoated Si AFM tip.

S7 Comparison of tip-only signal on SiO_2/Si and fused silica

Figure S7 compares the tr-SNOM signal acquired on the SiO_2 squares on the AFM calibration grating TGQ1, similar to the measurement presented in the main text in Figure 6. The two measurements were acquired with the same uncoated Si AFM tip and roughly the same optical alignment, right after one another. The dynamics of the 4th tapping harmonic σ_4 are shown acquired with a photon energy of 1.9 eV, after photo-excitation at 3.0 eV. The photon energies and fluences are the same as for the measurements presented in the main text. On TGQ1, the SiO_2 layer is $\sim 20\,\mathrm{nm}$ thick on a Si substrate, while the fused silica substrate is $\sim 300\,\mu\mathrm{m}$ thick. This figure serves the purpose to validate the determination of a dynamic contribution of the AFM tip on TGQ1, as done in the main text.
